# The long COVID research literature

**DOI:** 10.3389/frma.2023.1149091

**Published:** 2023-03-24

**Authors:** Alan L. Porter, Mark Markley, Nils Newman

**Affiliations:** Search Technology, Inc., Peachtree Corners, GA, United States

**Keywords:** long COVID, text analysis, tech mining, bibliometrics, COVID-19, research profile

## Abstract

While the COVID-19 pandemic morphs into less malignant forms, the virus has spawned a series of poorly understood, post-infection symptoms with staggering ramifications, i. e., long COVID (LC). This bibliometric study profiles the rapidly growing LC research domain [5,243 articles from PubMed and Web of Science (WoS)] to make its knowledge content more accessible. The article addresses What? Where? Who? and When? questions. A 13-topic Concept Grid presents bottom-up topic clusters. We break out those topics with other data fields, including disciplinary concentrations, topical details, and information on research “players” (countries, institutions, and authors) engaging in those topics. We provide access to results *via* a Dashboard website. We find a strongly growing, multidisciplinary LC research domain. That domain appears tightly connected based on shared research knowledge. However, we also observe notable concentrations of research activity in different disciplines. Data trends over 3 years of LC research suggest heightened attention to psychological and neurodegenerative symptoms, fatigue, and pulmonary involvement.

## 1. Introduction: Profiling the long COVID literature

As the pandemic wanes, the outpouring (over 1,000,000 articles) of research on COVID-19 slows. However, what is the research pattern for long COVID (LC), the “pandemic after the pandemic”? The aim of this study was to profile LC research to generate actionable research intelligence for researchers, clinicians, and policymakers. That should serve to accelerate the resolution of LC medical and other effects.

We cast this article as a “research profile” (Porter et al., 2002). The themes we address include:

➢ Striving to understand the body of research study focused on LC issues. We work to depict this domain in ways that help interested parties grasp key parts and see how they come together over time.
➢ Trying to depict ways that this research domain is coalescing. We seek to characterize major topics and key researchers in order to determine how they connect or remain largely separate.

Therein, the article develops a Dashboard to overview the research domain and provide components to help a user access particular research knowledge. The article digs deeper to get at, represent, and provide aids to access particular research findings in the domain.

Our research themes can be cast in terms of answering four of the so-called reporters' questions:

(1) *What* is being emphasized? [what topics are being studied?].
(2) *Where* is the work being done? [countries].
(3) *Who* is doing it? [the research community, i.e., disciplines, authors, and institutions; how do LC researchers connect; what are the networks?].
(4) *When*? [trends].

Of particular interest is to provide usable intelligence on combinations of those “4 W's,” e.g., to identify who is researching what sub-themes recently?

## 2. Background

“Research profiling” (Porter et al., 2002) uses “Text and Data Mining” (TMD) tools to gain perspective on a research domain. Such tools are growing increasingly powerful, drawing upon various artificial intelligence (AI) capabilities. These extend analyses from basic bibliometrics to probe more deeply into research content (Zhang et al., 2020). Enhanced computing power supports the development of text analytics to go beyond the study of terms separately to utilize contextualized term-to-term relationships, i.e., “term embedding” to improve clustering. Since 2019, embedding tools (e.g., word2vec and BERT) have advanced notably (c.f., Ethayarajh et al., 2019; Peters et al., 2019; Reimers et al., 2019).

The main tools we use for this research are rooted in “tech mining” (Porter and Cunningham, 2005; https://www.gtmconference.org/). This is shorthand for text analyses of Science, Technology & Innovation (ST&I) information resources to inform R&D management in various guises. Tech mining applies bibliometrics and text analyses of various sorts to gain usable research intelligence. A sampling of tech mining applications gives some feel for how this approach can help perceive research concentrations on given topics: nanotechnology systems of innovation (Miyazaki and Islam, 2007); term clumping for technical intelligence (Zhang et al., 2014); technology evolution pathways for 3D printing (Huang et al., 2017); and research profiling of nano-enhanced solar cells (Guo et al., 2010). Systematic reviews also have parallels with tech mining (Anderson et al., 2018). Here, we apply tech mining tools to profile the LC research domain.

Literature-based discovery (LBD) approaches extract intelligence on research concentrations within a domain to, then, identify pertinent research beyond the domain (c.f., Swanson, 1986; Swanson and Smalheiser, 1997; Smalheiser and Swanson, 1998; Kostoff, 2007). Enhanced data access enables LBD to be applied to entire databases, including PubMed (Wu et al., 2021). In our previous analyses of COVID-19 (Porter et al., 2020), we explored LBD-related techniques to help locate pertinent out-of-domain research. The present research sets the stage for LC LBD exploration, but does not undertake it.

Tracking ST&I topic evolution and key researchers in a research community is of potential interest (Glänzel et al., 2019). Such information is useful in pointing out new research opportunities, and it can help identify important contributors to the domain. It also helps map what constitutes a given domain and how that is evolving. Here, the spawning of a discrete LC domain out of the COVID-19 domain is of great interest.

Related tech mining themes include a depiction of science maps, science evolutionary pathways, technology roadmap, innovation pathways, and so on (c.f., Kostoff and Scaller, 2001; Rafols et al., 2010; Zhang et al., 2016, 2017). Applications of such research profiling have been directed at COVID-19 (Zhang et al., 2021) to help identify causes (Kostoff et al., 2021) and possible treatments (c.f., Kostoff et al., 2020).

Offering even greater potential for ST&I management would be forecasts of topics that are “emerging” (Robinson et al., 2013), i.e., topics accelerating in attention by the research community. We draw on the U.S. Intelligence Advanced Research Projects Activity (IARPA) Foresight and Understanding from Scientific Exposition (FUSE) Program efforts to extract ST&I intelligence, particularly, from full-text resources (e.g., full articles or patents; http://www.iarpa.gov/index.php/research-programs/fuse; also refer to Alexander et al., 2012). In addition, we draw on key conceptual aspects of “tech emergence” to identify a set of requirements and modes of research acceleration from Rotolo et al. (2015). Here, we use abstract records instead of IARPA-preferred full text and adapt thresholds to meet the criteria of term novelty, persistence, community, and growth (Carley et al., 2018; Porter et al., 2018). We also combine four trend measures to detect accelerating usage. We generate resulting “Emergence Indicators” *via* a set of calculations consolidated into a routine provided in *VantagePoint* software (www.theVantagePoint.com).

We note that others have profiled COVID research activity. Zhang et al. (2022) conducted bibliometric analyses of 5,329 COVID-19 Web of Science (WoS) publications related to neurological considerations. They treat “what, where, who, and when” issues, as do we here. Of special interest, they go on to examine seven topics in detail; one of which is LC. While they do not profile LC research *per se*, they probe symptoms and get into key findings of particular studies, focusing on neurological issues. They note that LC is a topic drawing increasing COVID-19 research attention. It is interesting to see how research profiles of an exponentially expansive domain are sensitive to time, data source(s), and search queries. Zhang et al. addressed some 5,000 articles; we (Porter et al., 2020) dealt with some 70,000 PubMed-indexed articles; current estimates suggest over 1,000,000 articles published that relate to COVID-19. This study analyzes some 5,000 LC publications.

Urru et al. (2022) profile COVID-19 literature for the period of November 2019 to December 2021. They analyzed a consolidated and cleaned set of 269,198 records from Scopus, PubMed, and WoS (merged). They also overviewed nine other analyses of the COVID-19 research literature, eight of which covered periods only through part of 2020 (one reaches up to February 2021). They extracted 357,781 terms from the abstract records and reduced those to 8,813 words appearing in at least 100 records. They ran structural topic modeling (STM) and chose a 10-topic cluster solution. Excerpting: The most popular topic was related to the clinical pictures of the COVID-19 outbreak, which has a constant trend, and the least popular includes studies on COVID-19 literature and databases. “Telemedicine,” “Vaccine development,” and “Epidemiology” were popular topics in the early phase of the pandemic; increasing topics in the last period are “COVID-19 impact on mental health,” “Forecasting,” and “Molecular Biology.” Our LC topic clusters (Section 5.3) differ, seeming rather more sharply defined; one could pursue whichever set has elements most related to one's interests. Urru et al. note an increase in *mental health* concerns in the LC corpus over time; that emphasis also reflects in our LC topics, which include neuropsychiatric symptoms, cognitive deficits, and neurological sequelae.

Jin et al. (2022) have profiled *LC research*, as do we here. They retrieved 784 articles from Scopus (inclusive of PubMed) through December 2021. Our current study analyzes 5,243 articles from PubMed (also, a subset of those retrieved from WoS) through November 2022. Therefore, our dataset is strikingly larger (some 7X) in less than one extra year of data. However, LC research is tiny compared to COVID-19 research, as noted. A few comparisons between Jin et al. (2022) and our present LC “what, where, who, when” tabulations give a sense of this growing domain:

➢ Publication rate, a rough comparison for July–September 2021, they show over 200; we show over 400, i.e., about double. This indicates query expansion (and we have noted that done by the National Library of Medicine for their designated LC query within our own searches over time).
➢ Top countries for articles published, i.e., Jin et al. vs. this study^1^: the US (117 vs. 1,317), the UK (74 vs. 151), and Italy (71 vs. 626).
➢ Top journals, i.e., their top five remain in our top six (refer to Section 4.5.1) given the ~seven-fold increase in our dataset, which shows surprising consistency. The one added by us is *Cureus*.^2^
➢ Top cited authors vary from our current results interestingly (they use Scopus; we use WoS; and we add some 11 months). Nalbandian et al. is #1 for them with 396 citations, up to 594, and #3 for us (Table 1). Greenhalgh is #2 for them with 365 citations and #6 for us with 314. Sudre is their #3 and our #5. Mandal is their #4 with 135 and our #23 with 195 citations.
Most interestingly, our #1 is Huang et al. (2021), with 789, but not in their top 10; and Carfi et al. (2020), with 730, is our #2, but not in their top 10. Differences surely reflect timing, given how the time span expands for us.
➢ Their explorations of topical emphases focus on keyword frequency, whereas ours offer topical categorization *via* Concept Grid (based on PCA), i.e., different perspectives.

**Table 1:** Citation of the three most-cited articles by each leading long COVID web of science category.

	**# Records**	**793**	**338**	**297**	**287**	**277**	**255**	**243**	**218**	**217**	**192**	**166**	**161**	**150**	**147**	**121**	**120**	**116**	**105**	**101**
**# Records**	**Web of Science Categories**	**Medicine, General & Internal**	**Immunology**	**Public, Env & Occup Health**	**Neurosciences**	**Clinical Neurology**	**Infectious Diseases**	**Medicine, Research & Exper**	**Respiratory System**	**Psychiatry**	**Cardiac & Cardiovascular Systems**	**Pharmacology & Pharmacy**	**Microbiology**	**Biochemistry & Molecular Biology**	**Multidisciplinary Sciences**	**Pediatrics**	**Health Care Sciences & Services**	**Environmental Sciences**	**Virology**	**Cell Biology**
789	Huang et al. (2021)	22%	17%	21%	17%	16%	27%	22%	33%	18%	15%	12%	25%	23%	18%	9%	19%	22%	20%	22%
730	Carfi et al. (2020)	20%	16%	22%	14%	12%	26%	14%	25%	6%	15%	13%	26%	19%	20%	12%	27%	25%	24%	17%
594	Nalbandian et al. (2021)	17%	15%	12%	15%	14%	19%	15%	12%	15%	16%	19%	18%	15%	14%	7%	20%	12%	13%	18%

Jin et al. (2022) also analyze citations by country cited and international (country) collaborations.

## 3. Data and methods

### 3.1. Data

Our search in PubMed on 15 November 2022, used the National Library of Medicine (NLM) standard LC search:

→ (covid) AND LitCLONGCOVID[filter].
→ at: https://pubmed.ncbi.nlm.nih.gov/?term=%28covid%29$+$AND$+$LitCLONGCOVID%5Bfilter%5D.

That search yielded 5,243 records with abstracts (out of 6,015). We downloaded these and conducted our analyses on them. The 5,243 PubMed IDs were available, so we entered those in a PubMed search and retrieved the record set for further analyses:

➢ COVID https://sites.google.com/searchtech.com/covidproject/home, or at.

➢ http://bit.ly/3iJ5OAt.

Someone seeking to extend these analyses could start with the query operations noted; then, adjust for record additions from the time of our search, as warranted. The data treatments described in Section 3.2 indicate how we refined the record content.

On 16 November 2022, we searched for those 5,243 records in WoS using PubMed ID, thereby retrieving 4,292 (some 82% of the PubMed set). For topical analyses, we used the PubMed data. The main purposes for also getting the WoS version of the same articles were to use Web of Science Categories (WoSCs) to study disciplinary involvement and to use WoS Cited Reference content to enable citation analyses. Essentially, all the 4,292 records have Cited References.

### 3.2. Data treatment

We utilized various *VantagePoint* tools to extract, clean, and consolidate the fielded data of the record sets. These computer operations, plus certain manual refinements, included:

➢ Processed date information to generate publication month for each article over the period of January 2020, through December 2022 (with November and December only partial) for 4,393 records. For example, some date variants were: “2021 Apr”; “2021 Apr 1”; and “2021 Apr–Jun 01.”
➢ Combined institution name variations (and consolidated department levels within an organization) to reduce 15,010 names to 10,497, of which 2,356 were associated with more than a single article. PubMed Affiliation names are dependent upon submitted data and therefore inconsistent. Manual curation was completed on institutions, generally on all institutions with more than five records in the dataset. Individual department or campus location was not considered, with the exception of the University of California system.
➢ Consolidated author name variations using a *VantagePoint* fuzzy matching routine tailored to person names. To illustrate, the second most active author was Patrizia Rovere-Querini; the “List Cleanup” routine combined 18 instances of her name in that format with six lacking the hyphen, one showing as “Querini, Patrizia Rovere,” and one as “Rovere Querini, P.”
➢ Extracted, using Natural Language Processing (NLP), 126,740 abstract noun phrases; applied *VantagePoint*'*s* “RefineNLP” set of routines to consolidate closely related term variations (e.g., stemming; applying various thesauri to remove “stopwords”). We further tuned the abstract NLP phrases by removing terms closely related to LC search terms (based on our judgment, e.g., remove “COVID-19”). These processes yielded a set of 97,996 noun phrases. To illustrate, two of the resultant leading phrases were “sequelae,” appearing 1,101 times in 949 records after treatment (vs. 586 times in 505 records prior), and “persistent symptoms,” appearing 410 times in 303 records after treatment (vs. 383 in 288 records beforehand).

**Figure 1:**
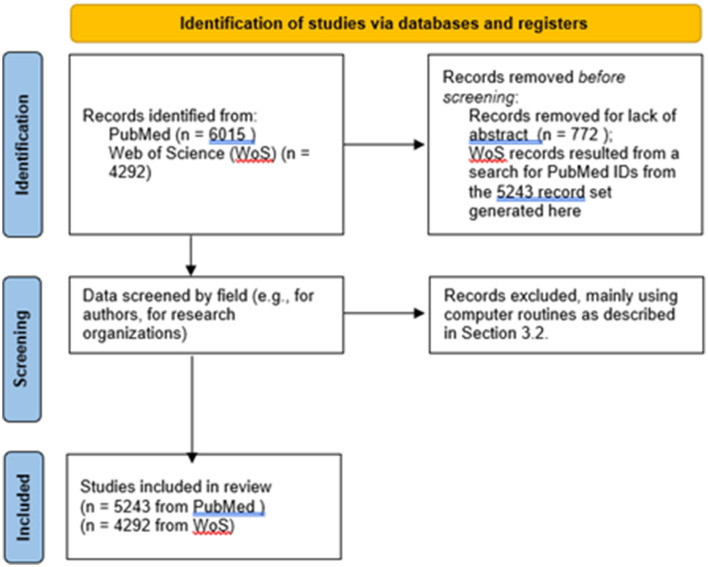
PRISMA flow diagram.

Figure 1 represents key data screening actions in a PRISMA diagram.

We considered various topical data resources, i.e., Medical Subject Headings (MeSH); keywords (WoS offers both author keywords and Keywords Plus, i.e., index terms automatically generated from the titles of cited articles); and title or abstract NLP-derived noun phrases. MeSH terms are pre-established and then applied to the set of articles under study. Here, we used them for some purposes but prefer a more adaptable set of terms deriving from the articles in our fast-evolving domain for “bottom-up” topic identification.

We also used the WoSCs for disciplinary characterizations; those are based on the journal (or conference) in which the articles appear, not on the article's content. We drew on abstract phrases, on the rationale that these were most prevalent and most fluid to pick up new research matter at the article level. Alternative topical data resources included Title NLP-derived phrases. We explored using these in conjunction with the Abstract NLP-derived phrases but went with the abstract NLP noun phrases in investigating this very new LC research domain.

Likewise, we treated the WoS 4,292-record dataset. Of particular note, we sought to mine the Cited Reference (“CR” field) information therein. As an illustration, here are a few references cited by J. Calvo-Paniagua et al. (e.g., Fernandez-de-Las-Penas, Cesar) in an article titled “A tele-health primary care rehabilitation program improves self-perceived exertion in COVID-19 survivors experiencing Post-COVID fatigue and dyspnea: A quasi-experimental study”:

➢ Jacobs L. G., 2020, PLOS ONE, V15, doi: 10.1371/journal.pone.0243882.
➢ Kendrick K. R., 2000, J Emerg Nurs, V26, P216.
➢ Lee K., 2009, GLOB INST, P1.

Note the sparsity of the cited record information. We obtained information of interest for our analyses on first author (with initials, not full name) of the cited article; year of cited article publication; cited journal (or conference) name (abbreviated); and DOI, where available. We did not use the volume and page information. For our purposes, we elicited the cited (first) author, cited year, cited journal, and cited DOI. We used a thesaurus to associate the cited journal (or conference) names with their corresponding WoSCs. This gave us a field of Cited WoSCs for further analyses (leading to strong indications of connectedness in the LC research domain).

### 3.3. Methods: Clustering terms into topics

Grouping-related variations of terms and phrases to determine meaningful topics are vital to comprehending latent themes in a body of text. There are a number of diverse methods to achieve such ends, including factor analyses, cluster analyses, and hierarchical analyses. Our colleagues have applied three such approaches to COVID-19 research to compare results and identify four promising research topics (Wu et al., 2022, and under revision).

In our LC case, the text consists of abstract records on biomedical research. We build our main topic analysis using NLP^3^ on noun phrases extracted from the PubMed abstracts. The 5,243 records yielded some 95,551 such terms and phrases, after refinement and cleaning from the original 126,740.

We ran the Principal Components Analysis (PCA) routine provided in *VantagePoint* as “Factor Map.” PCA is actually a basic, widely used form of factor analysis. This version is tailored to scientific text (e.g., protecting chemical formulas). In the past, we have compared topic modeling approaches to generate effective topic clusters on WoS abstract records (Yau et al., 2014). We compared PCA results to the Concept Grid option in *VantagePoint*. Concept Grid traces back to principal components decomposition (PCD). A key feature is the application of an optimization routine in conjunction with PCA to cover a maximum number of records with a minimum number of groups (factors). The resulting set contains fewer factors than our PCA solution, but those cover more of the records. The 13 Concept Grid factors have their constituent high-loading terms (above threshold) associated with 4,358 of the 5,243 records. In contrast, our favored PCA solution of 23 factors only covers 3,124 of the records.^4^

Concept Grid gives a reproducible solution^5^ for a given set of records. It standardizes term selection and the number of factors generated for a record set. Watts and Porter (1999) and Watts et al. (1999) devised and applied PCD (the predecessor basis for Concept Grid). PCD automatically derives a min-max problem solution. It determines the number of factors by minimizing the entropy and maximizing the cohesiveness of the derived factor groups. The routine seeks to include as many of the records as possible, to be represented in the factor set. It also seeks more factors and more high-loading terms for each factor. The algorithm minimizes the duplication of constituent abstract records among the factors (Watts et al., 2004).

**Figure 2:**
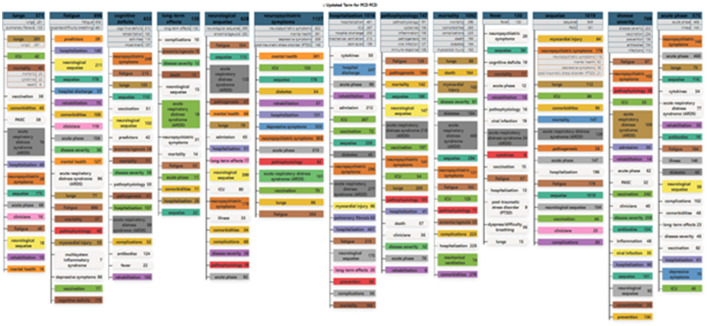
Concept grid of long COVID research topics.

We decided to apply the Concept Grid approach here. A key advantage is that it offers the Concept Grid (Figure 2, discussed later), which is an attractive asset in visualizing and exploring main topics and their subtopics. Both this and the PCA approaches reduce the 95,000 NLP phrases to 10–20 or so topics.

We ran Concept Grid on the record-term matrix (for abstract NLP phrases); it yielded 13 factors. We proceed to use these for our topical analyses to follow.

We used proprietary software, *VantagePoint* (www.theVantagePoint.com), to perform a number of text cleaning and consolidation steps, as well as analytical operations. Its RefineNLP routine consolidated abstract NLP phrases in preparation for topical analyses. Other software (e.g., *MS Excel, R*) can do many of the analyses. The open-access Dashboard [https://searchtechnology.github.io/LongCovidDashboard/] provides many “what, where, who, and when” results in a form suitable for zooming in on particulars. Some analyses requiring *VantagePoint* are:

➢ NLP routine has been tailored to scientific text; generally speaking, differences from other NLP routines should not be excessive. Note that we process noun phrases, including single-word ones.
➢ Emergence indicators [steps are delineated in Carley et al. (2018) and Porter et al. (2018)].
➢ Concept Grid (a variant of PCA with optimization routine).
➢ Cross-correlation map (**Figure 5**) based on WoSC co-citation of journals.

## 4. Results

### 4.1. Long COVID dashboard

**Figure 3:**
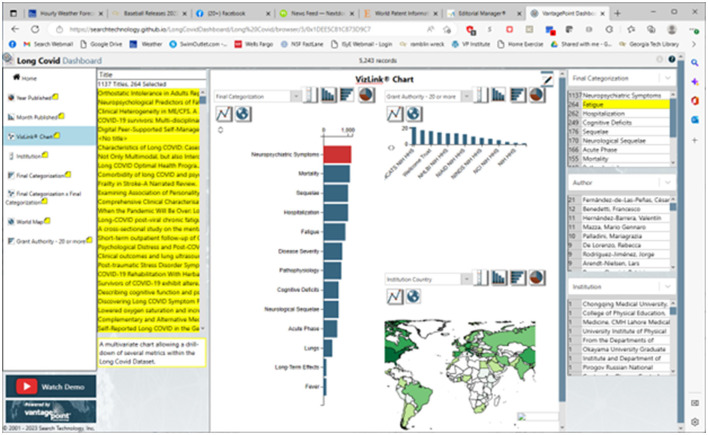
The long COVID dashboard: Sample VizLink^®^ view.

We have posted a dashboard presenting a research profile overview: https://searchtechnology.github.io/LongCovidDashboard/. We invite those who are interested to visit this site where we suggest “Watch Demo,” a 2-min YouTube walkthrough link. It introduces the world map, showing LC publication concentrations by country; another view shows the trend in publications. From a given view, three Detail Windows, on the right side (refer to Figure 3), associate with a selected target of interest to break out corresponding information.

Figure 3 shows the Dashboard of 5,243 PubMed LC records opened to the VizLink^®^ Chart view on 18 November 2022. In Figure 3, we arbitrarily clicked on the “Neuropsychiatric Symptoms” to see 1,137 of the records associated with those. When we look at the “Final Categorization” detail window (upper right), we might note that 264 of those records also treat fatigue. By clicking on those, we highlight their titles in the left title window. When we double-click any one of those, we open the abstract record where we could link to the active PubMed URL to read the article.

The Dashboard intends to help one identify research of interest. It offers “handles” to focus on particular fields and explore combinations of them:

➢ What: the 13 topical categories.
➢ Where: country or institution.
➢ Who: author.
➢ When: selection by year or month published.

### 4.2. Long COVID basic demographics

#### 4.2.1. Long COVID publication trend

**Figure 4:**
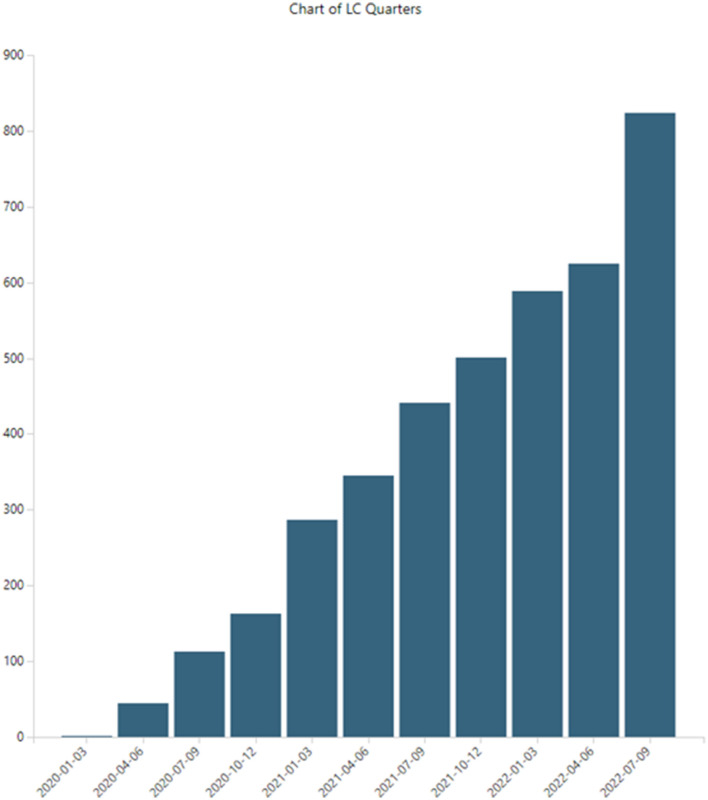
Long COVID publications.

LC research is growing. Figure 4 shows PubMed LC publications by 3-month periods (search performed on 15 November 2022; hence, fourth quarter, 2022, is incomplete and not shown here, although it is included in the 5,243 datasets and in 6-month breakouts, like **Table 7**). Publications indexed in PubMed increased from two articles in the first quarter, 2020, to 824 in the third quarter, 2022.

This is not unexpected growth for LC, given the massive growth in the overall COVID-19 numbers. Historically, overall growth rates in scientific publications amount to 4.1% with a doubling time of 17.3 years (Bornmann et al., 2021). In our previous COVID-19 study (Porter et al., 2020), we identified hyper-exponential growth in articles in PubMed. Starting from 199 in January 2020, we observed over 41,000 articles containing unique abstracts for the year. In 2021, that total number more than doubled to over 85,000 articles. Teixeira da Silva et al. (2021) tallied some 23,634 COVID-19 articles for the first half of 2020, searching in WoS and Scopus, quite in line with our 2020 tally. More recently, Nane et al. (2022) modeled COVID-19 publication growth using the broader Connections dataset, tabulating 464,217 as of 31 May 2021, and projecting nearly 900,000 by 27 March 2022.

Note that the LC publication set is impressive, building to over 5,000 articles in <3 years. However, it is dwarfed by COVID-19, with over 1,000,000 articles.

#### 4.2.2. Disciplines engaged

As indicated in Section 3.1, “Data,” we downloaded 4,292 WoS records that correspond to the 5,243-record PubMed search and could be found in a WoS “PubMed ID” field search (82% of these PubMed articles found indexed in WoS).

Here, we present publication WoSCs information as an indicator of which disciplines are researching LC-relevant issues. WoSC classification is based on the Clarivate categorization of the journal or conference, not on the individual article content.^6^ It incorporates both journal (conference) cross-citation patterns and expert judgment on research domains. Some journals (conferences) appear in more than one WoSC.

Disciplinary engagement of LC is strikingly broad. The 4,292 articles are associated with 126 WoSCs (i.e., half of the total of some 250 WoSCs); 104 WoSCs have two or more publications in the set (full list in the Supplementary Table S1). The leading WoSCs are:

**Table:** 

(1) Medicine, General & Internal	793
(2) Immunology	338
(3) Public, Environmental & Occupational Health	297
(4) Neurosciences	287
(5) Clinical Neurology	277

newline The strong involvement of “neural” in LC is prominent. Table 1 shows the 19 WoSCs with more than 100 LC articles as columns. Walking across the WoSCs, most cited these three articles to a similar degree. Exceptions include Respiratory, which accentuates Huang et al. (the Wuhan discharged patients 6-month Chinese assessment). Notably, low in citation propensity by Psychiatry is Carfi et al. (the Italian study of persistent symptoms in post-COVID-19 former patients). Again, breadth stands out. By simply scanning the list, we note multiple organ systems and ages, including pediatrics and elderly people (not shown, #29 with 49 articles).

### 4.3. Disciplines and citations

We gain further insight into LC research disciplinary engagement by examining citation information. WoS records provide limited Cited Reference information (recall examples in Section 3.1 and introduction in Section 3.2). However, these are of value to us at several levels to analyze (1) particular articles being cited (by using DOIs), (2) particular first authors cited, and (3) cited journals (conferences). As mentioned earlier, a journal-to-WoSC thesaurus provides (4) cited WoSC information.

Our 4,292 LC WoS articles yield 94,325 cited DOIs. The sense in looking at the list is of an *amazingly dense research knowledge network*, e.g., 69 DOIs are cited by 100 or more of the 4,292 LC articles. In this very recent research domain, which spans so many disciplines, we might have hypothesized a scattered and minimally shared pattern of referencing. We present three approaches to give a sense of the nature of the LC research knowledge base.

#### 4.3.1. Three most-cited articles by long COVID research

Three articles are cited especially highly [each of these by ~14%−18% of this very multidisciplinary dataset: Huang et al. (2021) with 789 citations, Carfi et al. (2020) with 730 citations, and Nalbandian et al. (2021) with 594 citations—their foci]:

➢ Huang et al.: 6-month consequences of COVID-19 in patients discharged from hospital in Wuhan (China).
➢ Carfi et al.: Persistent symptoms in 143 post-COVID outpatient clinic patients (Italy).
➢ Nalbandian et al.: A review of the LC literature, proposing a multidisciplinary care approach with dedicated COVID-19 clinics (USA).

We note that these three articles are foundational to LC and are likely cited as background references. Huang and Carfi are early case studies describing post-acute symptoms in patients with COVID-19, and the Nalbandian article is an LC literature review. We checked to learn that this LC search captures only about half of the total citations to the three articles in the WoS Core Collection. The three articles' focus is on COVID, not limited to LC. Furthermore, all three are very recent (2020 or 2021) to have accrued so many citations; it is an energetic research community!

Table 1 shows the distribution of citing of these three articles by the 19 LC publication WoSCs with more than 100 articles. These 19 WoSCs account for some 79% of the 4,292 article set. The rows in Table 1 show the percentage of a given WoSC's LC articles that cite that article. Amazingly, 18 of the 19 WoSCs show at least 12% of their articles citing each of the three articles. This attests to the breadth of engagement within LC. To give the flavor of the disciplinary span, in the 4,292 records, 12% or more of the articles in Public Health, Clinical Neurology, and Cell Biology all refer to the three articles.

#### 4.3.2. Long COVID research: Leading Web of Science Categories by leading cited WoSCs

Table 2 shows the theme of the commonality of citation over the LC record set. Here, the columns again show LC publication activity by the 19 leading WoSCs. The rows show the cited WoSCs (based on cited journals or conferences receiving 200 or more citation instances in the dataset). Note the remarkable extent of cross-disciplinary citation. As an example, the highlighted column for Psychiatry is scanned, a field that could be considered falling somewhat apart from mainstream biomedical science. For Psychiatry's 217 articles in this LC record set, a median of 36% of those articles refer to articles appearing in the given WoSCs. For instance, 92% of LC articles published in a Psychiatry journal/conference cite something from a General Medicine journal/conference (that is actually a bit more than 88% that cites something from Psychiatry). At the other extreme, we might say that Pediatrics and “Rehab” stand apart from each other; only 2% of Pediatrics articles in the set cite a Rehabilitation journal/conference. However, the message lies not in such details; it is that the LC research literature is remarkably multidisciplinary in both where it is published and in the research knowledge upon which it draws (citations).

**Table 2:** Main long COVID publication Web of Science Categories (WoSCs) by the WoSCs of the articles they cite.

			**1**	**2**	**3**	**4**	**5**	**6**	**7**	**8**	**9**	**10**	**11**	**12**	**8**	**14**	**15**	**16**	**17**	**18**	**19**
		**# Records**	**793**	**338**	**297**	**287**	**277**	**255**	**243**	**218**	**217**	**192**	**166**	**161**	**150**	**147**	**121**	**120**	**116**	**105**	**101**
**1**	**# Records**1	**Web of science categories: based on Journals of publication (columns) by Citations received (rows)**	**Medicine, General & Internal**	**Immunology**	**Public, Env & Occup Health**	**Neurosciences**	**Clinical Neurology**	**Infectious Diseases**	**Medicine, Research & Exper**	**Respiratory System**	**Psychiatry**	**Cardiac & Cardiovascular Systems**	**Pharmacology & Pharmacy**	**Microbiology**	**Biochemistry & Molecular Biology**	**MultidisciplinarySciences**	**Pediatrics**	**Health Care Sciences & Services**	**EnvironmentalSciences**	**Virology**	**Cell Biology**
2	3922	Medicine, General & Internal	92%	93%	94%	92%	92%	94%	95%	92%	92%	90%	93%	94%	90%	90%	90%	93%	96%	95%	91%
3	2686	Multidisciplinary Sciences	55%	83%	65%	71%	65%	68%	77%	46%	64%	53%	76%	83%	90%	77%	45%	63%	65%	74%	94%
4	2618	Infectious Diseases	59%	79%	60%	64%	60%	83%	70%	58%	54%	48%	66%	86%	73%	65%	65%	52%	62%	72%	68%
5	2595	Immunology	51%	90%	54%	69%	65%	75%	79%	44%	70%	40%	72%	84%	83%	62%	69%	45%	57%	76%	84%
6	2539	Medicine, Research & Experimental	54%	83%	52%	62%	58%	65%	83%	48%	58%	48%	69%	77%	83%	66%	46%	58%	52%	73%	89%
7	2135	Biochemistry & Molecular Biology	44%	78%	33%	60%	48%	55%	71%	37%	39%	44%	64%	71%	89%	59%	38%	41%	34%	68%	90%
8	2057	Respiratory System	53%	45%	45%	41%	35%	48%	46%	93%	34%	52%	53%	53%	49%	48%	31%	43%	47%	51%	50%
9	1994	Cell Biology	41%	74%	32%	53%	44%	52%	66%	36%	36%	43%	57%	69%	82%	53%	36%	40%	32%	67%	86%
10	1938	Microbiology	41%	70%	40%	47%	42%	64%	59%	37%	28%	30%	58%	76%	73%	53%	40%	28%	38%	66%	69%
11	1820	Critical Care Medicine	45%	38%	32%	41%	36%	36%	42%	83%	35%	52%	51%	44%	45%	39%	21%	34%	38%	38%	45%
12	1705	Virology	32%	59%	32%	49%	45%	42%	50%	25%	34%	28%	57%	58%	72%	46%	26%	28%	35%	73%	61%
13	1536	Clinical Neurology	32%	27%	33%	92%	91%	27%	37%	14%	69%	20%	35%	31%	44%	30%	23%	26%	41%	29%	35%
14	1525	Cardiac & Cardiovascular Systems	40%	27%	27%	25%	19%	29%	36%	62%	17%	89%	39%	30%	39%	32%	25%	26%	30%	27%	36%
15	1470	Neurosciences	29%	30%	32%	90%	88%	30%	39%	14%	67%	14%	36%	35%	42%	29%	18%	24%	42%	35%	35%
16	1436	Biology	28%	37%	43%	42%	34%	35%	40%	24%	41%	27%	43%	42%	49%	39%	18%	38%	46%	33%	48%
17	1393	Public, Environmental & Occupational Health	32%	29%	62%	27%	27%	39%	34%	22%	43%	20%	27%	34%	32%	36%	39%	47%	72%	32%	25%
18	1214	Psychiatry	23%	18%	42%	66%	66%	24%	23%	17%	88%	8%	27%	18%	27%	29%	15%	33%	46%	21%	21%
19	1006	Hematology	21%	31%	12%	21%	15%	19%	34%	18%	8%	66%	25%	20%	41%	18%	21%	8%	14%	25%	39%
20	953	Peripheral Vascular Disease	21%	21%	11%	25%	22%	16%	31%	17%	9%	66%	25%	16%	35%	17%	22%	8%	12%	20%	33%
21	732	Radiology, Nuclear Medicine & Medical Imaging	16%	12%	8%	30%	25%	15%	14%	36%	11%	38%	16%	17%	19%	14%	12%	12%	6%	11%	12%
22	616	Sport Sciences	15%	7%	19%	16%	13%	11%	12%	23%	8%	15%	11%	9%	9%	10%	4%	18%	29%	12%	8%
23	495	Rehabilitation	13%	5%	16%	13%	12%	11%	9%	20%	8%	6%	10%	9%	7%	10%	2%	13%	24%	12%	8%
	467	Pediatrics	9%	13%	9%	11%	10%	12%	9%	6%	8%	11%	8%	9%	13%	4%	83%	5%	12%	10%	9%
		MEDIAN	32%	37%	33%	47%	42%	36%	40%	36%	36%	40%	43%	42%	45%	39%	26%	33%	38%	35%	45%

This widespread commonality of citation suggests a well-connected research domain. The contrary finding would have been “islands” of activity unto their own. Based on the degree of shared research knowledge reflected by cited references, we do not see that.

#### 4.3.3. Long COVID research: Mapping Web of Science Categories based on similarity in cited WoSCs

The prior citation analyses should not imply that LC research presents a singular, fully sharing domain. For example, articles in one WoSC do not uniformly and heavily cite all others. In Table 2, Psychiatry publications are highlighted; note that several WoSCs are rarely cited by Psychiatry articles (e.g., Hematology).

**Figure 5:**
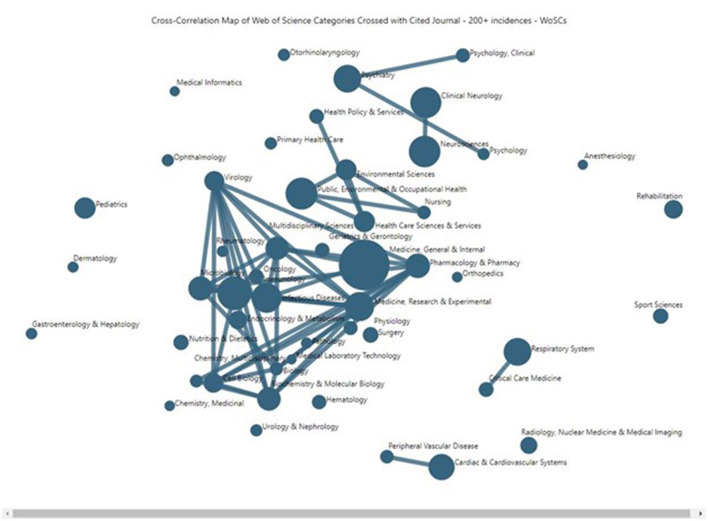
Long COVID publications' Web of Science Categories, mapping similarity in their citing of journals.

Figure 5 plots a more encompassing set of the top 50 LC publication WoSCs (whereas Tables 1, 2 more selectively addressed only the top 19 of those). This is a *VantagePoint* cross-correlation map reflecting the degree of affinity, based on the degree of similarity of the 49 WoSCs whose journals are cited at least 200 times (using instance counts) by the 4,292 articles (more encompassing than the top 23 cited WoSCs used in other analyses here). Larger nodes indicate more associated articles. The location along the *X* and *Y*-axes has no particular meaning in this representation. Nodes located nearby tend to be related. The more powerful indicator of relationship is the strength of lines shown connecting nodes (the software allows one to change the thresholds for the strength of association for connecting lines shown).

Figure 5 suggests that LC research has a heavily interconnecting core, with some major secondary concentrations of LC research, and a number of outlying, relatively separate subdomains. The LC research core includes some 20 or so WoSCs that are highly interconnecting in the central area of the map, including:

➢ Pharmacology & Pharmacy
➢ Multidisciplinary Sciences
➢ Virology
➢ Medicine, Research & Experimental
➢ Microbiology
➢ Biochemistry & Molecular Biology
➢ Immunology
➢ Infectious Diseases

Also, the “Medicine, General & Internal” node stands forth as the largest node and is quite central in Figure 5, scanning the top row of Table 2; over 90% of the articles in every one of the 19 sub-domains (publication WoSCs) cite one or more articles published in a journal categorized in the “Medicine, General & Internal” WoSC.

The relatively separate sub-domains include a number of single nodes, plus *Psychology*-related (triple nodes, near the top of Figure 5), *Neurology* (two nodes, nearby Psychology), *Public Health* and related (five nodes, simply above the central core), *Respiratory* (two nodes, lower right), and *Cardiovascular* (two nodes, bottom). This attests to the span of issues and research attendant to LC.

### 4.4. Long COVID research network based on co-citation

Tables 1, 2 and Figure 5 portray a well-connected LC research community. We now take another perspective, examining the community based on first author co-citation. Might there be a core body of highly cited researchers? If so, can we glean insights into the disciplines involved, key players, and degree of connectedness?

After modestly consolidating cited author name variations (applying *VantagePoint*'*s* list cleanup routine using the “person name” rule set), we reduce 81,558 to 78,453 researchers. We then pull out “Anonymous” and institutional authors (e.g., World Health Organization, Centers for Disease Control and Prevention) and examine 107 cited authors with 100 or more citations received.

We generated an auto-correlation map (Supplementary Figure S1) for those 107 highly cited authors. We opened a Detail Window to break out the WoSCs most often citing particular authors. We also generated a cross-correlation map (Supplementary Figure S2) that incorporates a degree of second-order relationship. So, if Author A and Author B are not cited by the same article, but they both are co-cited with Author C, this connection is figured in.

The heaviest extent of citation and co-citation comes from articles published in WoSC “Medicine, General & Internal.” That is the case for the three most-cited authors analyzed above, i.e., Huang, Carfi, and Nalbandian. Supplementary Figure S2 shows the frequency of WoSCs citing Carfi; General Medicine shows 161, followed by 66 from Infectious Diseases and 65 from Public Health.

Browsing the auto-correlation map, nearly all the highly cited authors appearing in the left half of Supplementary Figure S1 are most cited by General Medicine.

More interesting is to spot authors highly cited by other WoSCs, as a secondary indicator of what disciplines are pursuing LC research. Some examples (Supplementary Figure S1) show the WoSCs citing Mao (upper right of the figure), which is led by Neurosciences. Similar emphases appear for six of the seven in that upper right cluster, with the one modest exception, Lechien, most cited by Clinical Neurology, with Neurosciences the second most. Of note, WoSCs for citing the studies of these LC authors (not discernible in Supplementary Figures S1, S2, as we picked one node to show in the Detail Window) include the following:

➢ *Neurosciences* (Mao and others, upper right).
➢ *Psychiatry* (Kroenke, upper right; also Roges and Mazza, center top).
➢ *Immunology* (Hoffman, far right).
➢ *Cardiovascular* (Pentmann, center; connecting strongly with Shi, right).
➢ *Respiratory System* (Hui and others, center and lower left-center).
➢ *Pediatrics* (Buonsenso, lower left).

This offers a perspective on what research fields are addressing LC issues based on *commonality in authors cited*, compared to the last paragraph of Section 4.3.3, which addressed WoSCs based on *commonality in journals cited*. But, most essentially, Table 2 shows the 19 WoSCs in which most LC research is published (columns) by the WoSCs that are most cited. All of these concur in identifying General Medicine as the primary “home,” but we observe a substantial diversity of other disciplines engaged. The general sense (Table 2) is that these disciplines are talking to each other about LC issues.

### 4.5. Long COVID domain characteristics

We briefly discuss some other features of the LC dataset. Unless otherwise noted, we profile the 5,243 PubMed record set.

#### 4.5.1. Where is LC research published?

The 5,243 LC articles appear in 1,803 publications, led by these nine with over 50 articles each.^7^ The following are the numbers of LC articles and their journal impact factors (JIFs):

**Table:** 

Journal in which Published	# Publications	JIF
➢ *Cureus*	126	–
➢ *International Journal of Environmental Research and Public Health*	116	3
➢ *Journal of Clinical Medicine*	94	5
➢ *Frontiers in Immunology*	81	8
➢ *PLOS ONE*	64	4
➢ *Frontiers in Medicine*	63	5
➢ *medRxiv: The Preprint Server for Health Sciences*	58	–
➢ *Scientific Reports*	52	5
➢ *BMJ Open*	51	3


Browsing through the top 50 of these journals, two-thirds are overtly medical. The publication outlets are thus heavily biomedical, but somewhat diverse.

Shifting to WoS for citation information, the 4,292 articles most heavily cite leading medical journals (showing here the instances of an article being cited for the leading seven journals, with at least 2,000 citations):

**Table:** 

Cited Journal	#Citations	JIF
➢ *New England Journal of Medicine*	4,770	176
➢ *Lancet*	4,405	203
➢ *JAMA—Journal of the American Medical Association*	3,590	157
➢ *Nature*	2,658	70
➢ *Nature Medicine*	2,481	87
➢ *PLOS One*	2,333	4
➢ *BMJ—British Medical Journal*	2,273	96

It is interesting to see only one journal in common in the journals in which LC researchers publish most and those that they cite most, i.e., *PLOS One*. The JIFs for these journals with the most LC publications are quite respectable (i.e., LC is being published in reasonably strong impact outlets); the JIFs for the heavily cited journals are extraordinarily high, excepting *PLOS One*, raising some questions about JIF as a screen for importance, at least for LC research.^8^

#### 4.5.2. Leading countries

**Figure 6:**
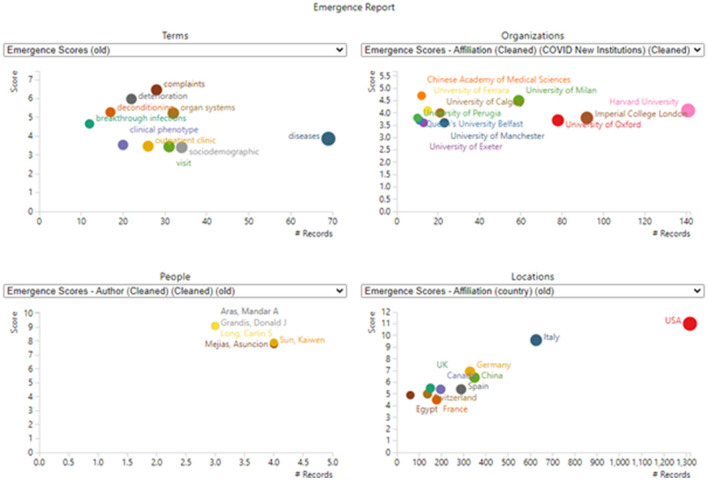
Emergence dimensions. Only the top entities in each section are shown (to avoid undue clutter). For example, 45 terms or phrases are scored as emergent (scoring over 1.77), but only 10 are plotted (scoring over 3.4). Values plotted reflect total counts in the dataset; they are not limited to records including emergent terms.

The US leads in LC publication with 1,317 of these 5243 articles, followed by Italy, China, Germany, and Spain. Figure 6 (lower right) shows the US leadership.

#### 4.5.3. Temporal patterns

The LC dataset concentrates on only 3 years; it shows strong growth:

**Table:** 

➢ 2020	408 articles
➢ 2021	1,913
➢ 2022	2,922 (for an incomplete year!)

Examining country publication rates for the leading five countries (Section 4.5.2) shows year-to-year growth for each. Growth from 2021 to 2022 is particularly strong in the European countries (Italy, Germany, and Spain, collectively, up from 389 to 689). The US and China show growth from 2021 to 2022, but not so strong (the US, up from 527 to 681; China, up from 139 to 167). Impressionistically, we hypothesized that the UK focus on LC was stronger than the US. The LC publication pattern in 6-month periods (c.f., Table 7) shows the UK as relatively sluggish compared to the US, e.g., no publications in the first half of 2020, rising to 26 in the second half of 2022. In contrast, the US shows nine articles in early 2020, rising to about 10-fold the UK rate in 2022 (292 in the first part; 288 in the second). Another marker of interest, i.e., Italy shows about half the US rate in 2022 (153 in the first half; 150 in the second). China's LC research also seems somewhat muted compared to its COVID-19 study, with 55 articles in the first half of 2022 and 62 in the second half.

Similar results appear if we consolidate countries into regions (Table 3).

**Table 3:** Long COVID publications for region by year.

	**# Records**	**408**	**1,913**	**2,922**
**# Records**	**4 Regions/year**	**2020**	**2021**	**2022**
1,872	Western Europe	110	648	1,114
1,487	North America	121	568	798
519	East Asia	47	195	277
239	South America	10	68	161

Further testament is that this research domain is fast-moving; the most-cited years for all articles receiving citations are 2020 (4,200 citations), 2021 (3,758), and 2022 (1,841, for a part-year). Given usual scientometric patterns that show lags for peak citation rates of a few years post-publication, this is impressive. It is reasonable, given that LC concerns follow the COVID-19 pandemic.

## 5. Exploring long COVID research topics

### 5.1. Emerging LC topics

Our “Tech Emergence Indicators” were introduced in Section 2; their rationale and construction are described by Carley et al. (2018) and Porter et al. (2018). In brief, a set of topical terms are prepared. Then, various thresholds are applied, and four *trends are combined* to distinguish topical terms that are notably accelerating in research attention, making a good case that these are cutting-edge topics in the domain (therefore, the basis of inclusion, detailed in the two articles noted, is based on a given term's pattern of occurrence over time; no judgment is involved in this). Here, these are calculated using a base period of January–March 2022, and an active period of April–October 2022. Therefore, this is a snapshot in time of topics being noted in the abstracts of LC articles published in that time frame that show accelerating research attention.

Table 4 presents an interesting mix of general medicine, broader health considerations, and specific medical issues. They also include some general terms that are not seemingly informative. Results are simply suggestive of topics increasingly drawing LC researcher attention during 2022. The counts are small (e.g., for the top term, “diseases,” parse 69 occurrences over 10 monthly time periods), so they are inherently not very stable.^9^ Also, monthly data, with low counts, are quite volatile.

**Table 4:** Highly emerging topical terms.

**Emergent terms**	**# Records**		
Diseases	69	Schools	23
Clinical practice	46	Deterioration	22
Medications	44	mRNA vaccines	22
Arrhythmias	43	Pulmonary disease	22
Health systems	39	Cardiopulmonary exercise testing (CPET)	21
Respiratory disease	35	Clinical phenotype	20
Sociodemographic	34	Thematic analysis	20
Physical health	33	Caring	19
Coronavirus	32	Daily activities	19
Inflammatory response	32	Online questionnaire	18
Organ systems	32	PFT	18
Visit	31	Pulmonary function tests (PFTs)	18
Health care systems	29	Recent evidence	18
Pericarditis	29	Deconditioning	17
Physical symptoms	29	Significant changes	16
Complaints	28	Tracheostomy	16
Outpatient clinic	26	Humoral immune response	15
Adverse effects	24	Mean time	15
Keywords	24	Small number	14
Preferred reporting items	24	Breakthrough infections	12
CPET	23	Randomized controlled trial	12
Large proportion	23	Acupuncture	8
Patient groups	23		

Figure 6 provides four derived representations based on the emergent topical terms.

(1) The upper left diagram shows select emergent terms with their emergence scores and the number of records in which they appear. “Diseases” show the most attention of these, 69 abstracts discussing these in the period of April–October 2022.
(2) The upper right diagram spotlights research organizations most actively using the emerging terms in their abstracts. Harvard is especially active.
(3) The lower left diagram spotlights active authors publishing on these “frontier” topics.
(4) The lower right diagram shows the countries that stand out on emergence score and the number of records.

### 5.2. Clustering the topics

As introduced in Section 3.3.1, we use an optimization routine drawing on PCA to generate a “Concept Grid” that depicts topical emphases in the 5,243 PubMed LC dataset. Figure 2 presents this clustering of the abstract (NLP) phrases into 13 topic groups (the columns). Under each are sub-clusters. Column 1 indicates that 371 of the 5,243 abstract records include a term associated with lung (pulmonary) issues. Just below the heading appears “lungs,” indicating that 281 of those records contain the term “lungs” *per se*. Scanning down the first column, we note that of those 371 lungs-related records, 48 concern “fatigue” as well. That points to a similar breakout in Column 2 of 896 records noting “fatigue,” whether or not they also include a lungs-related term. That said, “lungs” is also present in 77 of those fatigue-containing records.

We present the Concept Grid as a way to explore the dataset. It can suggest connections. By opening a Detail Window in *VantagePoint*, we can examine other variables in conjunction with one of the 13 clusters here, or with a sub-cluster. To illustrate, the following are some leading values associated with this sub-domain of *Fatigue* > *Lungs*, i.e., 77 records:

➢ Specific abstract phrases: lungs (59); dyspnea/difficulty breathing; fatigue (49).
➢ MeSH
  ° Descriptors: COVID-19 (58); Humans (58).
  ° Qualifiers: complications (35); etiology (15); diagnostic imaging (14).
➢ Journal: *PLOS One* (4); *Frontiers in Immunology* (3).
➢ Country: USA (18); China (9).
➢ Research organization: Columbia University (3).
➢ Author: Becquemont and 13 others (2).
➢ Year published: 2022 (47), 2021 (28), 2020 (2) ^**^notably current interest.
➢ Grant Authority: NIH > NCATS (National Center for Advancing Translational Sciences) (3).

There are many possible sub-domain combinations and explorations. The coloring is intended to facilitate cross-factor scanning, e.g., to help spot “*ICU*” under lungs, neuropsychiatric symptoms, hospitalization, pathophysiology, mortality, sequelae, disease severity, and acute phase!

For another instance, an endocrinologist might want to investigate “diabetes” in LC. We spot 34 records related to diabetes as a sub-topic under neuropsychiatric symptoms; 43 under hospitalization; and 164 under mortality.^10^

### 5.3. Breakouts of other variables with the 13 topic clusters

This section lays out several two-dimensional breakouts in conjunction with the 13 “Concept Grid” topic clusters. We note that in the software (*VantagePoint*), one can readily break out an additional dimension. Here, we hope, we array several of general interest. Here, we list the 13 topic clusters generated *via* the “Concept Grid” optimization routine, along with their record frequencies. One caveat, alterations in the term set presented to the Concept Grid routine, would lead to different clusters. These should be taken as one of many possible ways to separate, label, and relate the LC topical emphases.

**Table:** 

Topic clusters	# of Records
Neuropsychiatric symptoms	1,137
Mortality	1,092
Sequelae	1,019
Hospitalization	10,16
Fatigue	896
Disease severity	769
Pathophysiology	747
Cognitive deficits	632
Neurological sequelae	628
Acute phase	575
Lungs	371
Long-term effects	135
Fever	130

We might think in terms of four of the “Reporter's Questions,” i.e., *What? Where? Who?* and *When?*

If we address “*What?”* as the 13 topic clusters, we could examine those in conjunction with other *What?* variables. Table 5 selects 14 frequently occurring, interesting MeSH descriptors to illustrate. One can investigate further, e.g., suppose we note that Immunoglobulin G is especially linked to Disease Severity (33 out of 75 records). By checking (by opening a Detail Window) which institutions are publishing on this combination, we could see that four of the six leading research organizations are in China.

**Table 5:** Selected MeSH descriptors by 13 long COVID topic clusters.

		**1**	**2**	**3**	**4**	**5**	**6**	**7**	**8**	**9**	**10**	**11**	**12**	**13**
	**# Records**	**1,137**	**1,092**	**1,019**	**1,016**	**896**	**769**	**747**	**632**	**628**	**575**	**371**	**135**	**130**
**# Records**	**Mesh descriptors**	**Neuropsychiatric symptoms**	**Mortality**	**Sequelae**	**Hospitalization**	**Fatigue**	**Disease severity**	**Pathophysiology**	**Cognitive deficits**	**Neurological sequelae**	**Acute phase**	**Lungs**	**Long-term effects**	**Fever**
286	Quality of life	125	53	53	87	97	26	28	76	27	47	17	11	0
283	Prospective studies	59	57	52	118	68	36	24	43	31	32	22	6	8
217	Pneumonia, viral	56	52	46	49	18	29	24	22	23	15	18	0	5
173	Risk factors	38	65	43	54	38	29	16	11	12	27	10	2	3
118	Anxiety	106	11	10	22	20	9	7	20	9	11	1	1	1
107	Dyspnea	21	13	21	34	80	14	8	26	7	14	14	6	1
105	Depression	99	8	9	15	15	4	10	16	5	9	1	2	1
77	Longitudinal studies	24	12	14	26	17	12	8	13	9	18	3	2	1
75	Immunoglobulin G	3	5	9	3	7	33	14	4	6	6	5	1	0
73	SIRS	8	23	18	10	3	9	21	3	10	7	3	3	10
62	Vaccination	6	14	14	10	1	42	9	2	5	0	2	0	0
59	Child, preschool	11	11	13	11	9	9	8	6	7	3	4	1	6
59	Comorbidity	11	32	17	17	8	9	7	7	5	4	5	0	2
59	Cytokines	3	14	9	4	8	15	35	5	12	6	6	2	1

One breakout of interest is *What?* (13 topic clusters) by *Where?* (countries). Supplementary Table S2 arrays the countries vs. the 13 clusters. Of 138 countries, 129 appear in the table. One can do research on a given topic group in a given country.

Table 6 consolidates countries into four select groups to look for regional differences in LC attention. The overall impression is that research emphases in these four regions are generally comparable. Comparing North America and Western Europe, North America has a greater emphasis on sequelae in contrast to Western Europe which has a greater emphasis on hospitalization, fatigue, and cognitive deficits; interest in acute phase of East Asia appears somewhat less than that of Europe.

**Table 6:** Four country groups by 13 long COVID topic clusters.

		**1**	**2**	**3**	**4**	**5**	**6**	**7**	**8**	**9**	**10**	**11**	**12**	**13**
	**# Records**	**1,137**	**1,092**	**1,019**	**1,016**	**896**	**769**	**747**	**632**	**628**	**575**	**371**	**135**	**130**
**# Records**	**4 country groups\topic cluster**	**Neuropsychiatric symptoms**	**Mortality**	**Sequelae**	**Hospitalization**	**Fatigue**	**Disease severity**	**Pathophysiology**	**Cognitive deficits**	**Neurological sequelae**	**Acute phase**	**Lungs**	**Long-term effects**	**Fever**
1872	Western Europe	24%	20%	15%	24%	21%	15%	14%	16%	13%	13%	8%	2%	2%
1487	North America	20%	23%	25%	16%	16%	16%	17%	10%	14%	10%	6%	3%	2%
519	East Asia	23%	18%	20%	18%	14%	18%	15%	12%	10%	7%	11%	4%	3%
239	South America	23%	19%	16%	23%	17%	10%	15%	19%	18%	15%	9%	0%	3%

**Figure 7:**
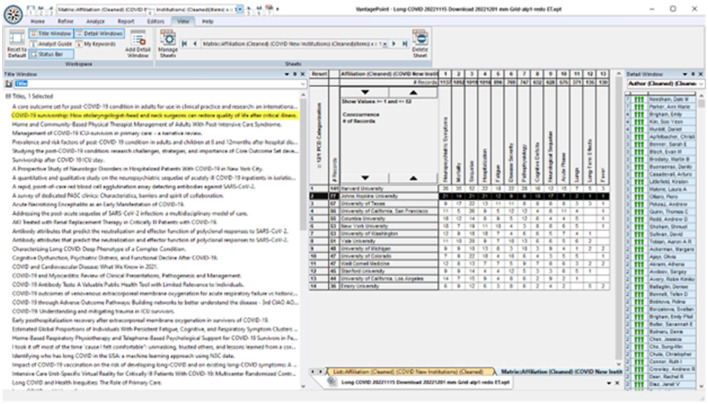
Exploring topics together with research players.

*Who?* is pursuing particular *What?* issues? We imagine exploring American universities emphasizing LC research on neuropsychiatric issues. Figure 7 breaks out data fields, including widespread engagement of neuropsychiatric symptoms (the first column of the 13 topical clusters) by 14 leading US universities. We probe more deeply to identify that two Johns Hopkins researchers – Dale Needham and Ann Marie Parker – are quite active, with seven articles each (right window). Next, we observe that one of Needham's articles is co-authored with Parker, titled “COVID-19 survivorship: How otolaryngologist—head and neck surgeons can restore quality of life after critical illness” (highlighted in the right window). We might open that abstract on how otolaryngologists—head and neck surgeons can restore quality of life. If we spot potential interests, we can click through to open the article itself (https://pubmed.ncbi.nlm.nih.gov/33545448/).

*When?* did the research target the 13 topics? The limited span of 3 years for LC research invites examination by month (as we used for partial 2022 in the emergence scoring of Section 5.1). LC research increased quite linearly from two articles dating in January 2020 to 290 in September 2022 (284 for October, and fewer in the following months, as of our November search date).^11^

Table 7 shows the percentage of a given 6-month period articles addressing each topic (Many articles address multiple topics.). Shifts in focus during this 3-year period seem to be relatively moderate. For these six 6-month periods, a topic that declined somewhat in relative emphasis^12^ is Mortality (that might be good news!). Topics that increased as a percentage of all in the 13 topics are Fatigue, Cognitive Deficits, Acute Phase, and Long-Term Effects.

**Table 7:** 6-Month periods by 13 long COVID topic clusters.

		**1**	**2**	**3**	**4**	**5**	**6**	**7**	**8**	**9**	**10**	**11**	**12**	**13**
	**# Records**	**1,137**	**1,092**	**1,019**	**1,016**	**896**	**769**	**747**	**632**	**628**	**575**	**371**	**135**	**130**
**# Records**	**Date published**	**Neuropsychiatric symptoms**	**Mortality**	**Sequelae**	**Hospitalization**	**Fatigue**	**Disease severity**	**Pathophysiology**	**Cognitive deficits**	**Neurological sequelae**	**Acute phase**	**Lungs**	**Long-term effects**	**Fever**
46	1-Jan-Jun, 2020	28%	28%	22%	26%	9%	11%	13%	22%	13%	9%	7%	0%	4%
275	2-Jul-Dec, 2020	22%	26%	21%	18%	7%	15%	13%	5%	12%	3%	8%	1%	4%
632	3-Jan-Jun, 2021	20%	21%	18%	17%	14%	13%	13%	10%	11%	9%	8%	3%	4%
942	4-Jul-Dec, 2021	18%	21%	21%	22%	18%	14%	15%	10%	11%	12%	8%	3%	2%
1213	5-Jan-Jun, 2022	23%	20%	20%	23%	18%	14%	14%	13%	13%	12%	7%	3%	2%
1285	6-Jul-Dec, 2022	23%	20%	19%	17%	19%	15%	12%	14%	11%	12%	5%	3%	1%

On a more granular level, we compare topical terms' prevalence from 2020 (the first two 6-month periods = 321 records) to July–December 2022 (the most recent ~6-month period = 1,285 records) to see “what's new?”, i.e., we are looking for particular terms of potential interest that first appear after the early period of research. Table 8 offers a list of terms appearing in 7 or more records in the July–December 2022 period that were absent from the 2020 records.

**Table 8:** Long COVID topical terms appearing in July–December 2022 (absent in 2020).

**PASC**
Adolescents
Biomarkers
PCC
PCS
Executive function
Gastrointestinal
Suffering
Oxidative stress
Daily activities
Hypercoagulability
Kidney
Myalgic encephalomyelitis/chronic fatigue syndrome (ME/CFS)
Omicron variant
Delta variant
Gastrointestinal symptoms
Muscle weakness
Outpatient clinic
Pericarditis
Body mass index
Breakthrough infections
Hypoxia
Muscle strength
PACS
Schools
Spirometry
Abdominal pain
Carbon monoxide (DLCO)
Cardiopulmonary exercise testing (CPET)
Chronic diseases
Chronic inflammation
Hospital anxiety
long-term complications
ME/CFS
PFT
POTS
Arthralgia
Autonomic dysfunction
Broad spectrum
Coagulation
CPET
Diet
Immune dysregulation
Molecular mechanisms
mRNA vaccines
Natural infection

## 6. Discussion

This article profiles the research on LC, as of November 2022. A prime target is to apply tech mining (combining intelligent bibliometrics and text analyses) that can facilitate locating LC research of interest. We have illustrated a few ways that the “Concept Grid” enables connecting data fields. The article shows ways that topic clusters can be counterposed with other variables, e.g., research organizations, to address “Who is studying What?” We welcome inquiries to help track specific interests in the LC dataset.

Long COVID presents a unique challenge, a sweeping array of impacts, massive global spread, and extreme uncertainty about the mechanisms whereby COVID-19 induces LC. In addition, this “pandemic after the pandemic” confronts a weary world. Therefore, the responsiveness of research funding^13^ and engagement of researchers are vitally important.

The article focuses on the 13 topic factors offered by the Concept Grid. Those spotlight 13 data-driven themes that are not constrained to prior topical structures. We break out several data fields in conjunction with those 13 topics. Table 5 gives MeSH descriptors for more detail on topics. Table 6 presents relative topical emphases by four geographical regions. One informal hypothesis we pursued was that the UK was directing way more attention to LC than the US; our data did not support that (e.g., through June 2021, we show 18 UK-authored articles vs. 272 US-authored). Table 7 tabulates shifts in topical prominence over 6 half-year periods. From the early LC research in 2020 to that in 2022, we observe the increasing emphasis on fatigue, cognitive deficits, acute phase issues, and long-term effects.

One can break out other data fields by the 13 topics by using the Dashboard (https://searchtechnology.github.io/LongCovidDashboard/). The Dashboard provides multiple perspectives on the LC research domain for easy, active exploration. It offers ready visual renditions of publication trends, topic categories, and leading countries and funders. The VizLink^®^ allows quick focus on intersections of the data fields. For instance, to explore very recent studies on LC and lungs, we could select 177 records for November/December 2022, choose the lungs category, and browse those nine titles. For one or more titles of interest, we can open and read the abstract records. For an article that is of keen interest, we can click on the PubMed URL provided to get the article “at your fingertips.” We intend to update the LC Dashboard as frequently as we can.

A key question for this research profiling is to ascertain the nature of the LC research domain (if it even warrants being considered a domain). Our interpretation is that there is, indeed, an LC research domain. It shares research knowledge to a notable extent for a highly multidisciplinary area of study (c.f., Tables 1, 2). Table 2 shows the LC domain to be impressively multidisciplinary in terms of both where articles are published and what articles are cited. The LC domain is substantial, over 5,000 articles, based on the NLM LC query of PubMed. However, it is tiny in contrast to the “parent” COVID-19 domain of more than 1,000,000 articles.

We have noted the breadth of shared citations across disciplines addressing LC. We cannot speak of how those references are treated by the articles citing them. For example, in the Wuhan study, Huang et al. (2021) suggested how to set the stage for issues related to COVID-19. We note that the top three cited articles receive about half of their citations from our LC publications; so, half of the citations drawing heavily on these three are outside the LC research domain (i.e., addressing other COVID-19 issues).

On the one hand, as noted, the LC research domain appears amazingly dense, i.e., tightly networked and heavily citing the same articles. The 4,292 WoS LC articles cite 69 articles 100 or more times. Thus, researchers treating LC topics share references in common to a striking degree, making for a body of research knowledge that is not separated into “silos.” On the other hand, Table 2 shows that such a multidisciplinary field has quite distinct disciplinary emphases. Figure 5 shows prominent disciplinary concentrations based on co-citation by the LC article set.

The article explores which disciplines (based on WoSCs) are engaging in LC-related research. One set based on citing LC work (Section 4.4) gives a feel for the breadth: neurosciences, psychiatry, immunology, cardiovascular, respiratory system, and pediatrics. Table 2 gives more information by crossing the 19 WoSCs having the most LC publications against the top WoSCs of the journals they cite.

The span of LC research shows in the 19 WoSCs with over 100 articles (Tables 1, 2) ranging over:

➢ Basic biomedical processes (microbiology, biochemistry and molecular biology, and cell biology),
➢ Multiple organ systems beyond respiratory (neural and cardiac),
➢ Different populations [pediatrics and elderly people (49 articles)],
➢ Broader issues (public, environmental, and occupational health; environmental sciences; multidisciplinary sciences).

With such disciplinary breadth of the LC research domain, it is surprising to see that research shows a high degree of citation “connectedness.” While citation concentrations certainly vary by discipline (Table 2), they do not present a sense of separate “silos” of disconnected research concentrations.

We also analyze specific topical phrases that appear as “emerging”. Table 4 lists those as suggestive, but they would vary if the time span or the input terms were modified. Figure 6 shows leading players, i.e., authors, organizations, and countries, with the US prominent. Table 8 provides a different way to get at novel LC sub-topics.

We duly note important limitations. We used the “official” LC query devised by NLM to identify relevant research. For an emerging research domain with especially amorphous boundaries, there are many conceivable alternative formulations that would yield different datasets. LC diagnosis is not clear-cut; the definition of what research on diverse organ systems and symptoms belongs “in” the domain is hard. LC research invites tools, such as LBD, to scout out to identify pertinent research outside a given set of research bounds.

Our analyses are of a “snapshot in time,” the 5,243 PubMed articles as of 15 November 2022. We note that the prior NLM query applied a month or so earlier yielded a dataset less than half the size due to rapidly advancing research and, more so, adjustment of the query to adapt to evolving research patterns. We also analyzed the corresponding 4,292 WoS records. Special COVID-19 compilations (e.g., CORD-19); other databases, such as Scopus; and other data forms (e.g., ClinicalTrials.gov) offer additional information resources worthy of exploration. They would surely present different perspectives on LC.

We welcome inquiries about particular interests that we might help you pursue. Future research, above all, warrants tracking how LC evolves. Conducting literature-based discovery (LBD) holds particular appeal. We explored the use of a knowledge model for vaccination regarding COVID-19 (Wu et al., 2022) that extended outside the COVID research domain to search all PubMed documents for pertinent work. Analogously, it would be interesting to pursue one or more LC topics (e.g., one of the 13 Concept Grid clusters), or terms, *via* formulation of a knowledge model that sets up 50 top and 50 bottom TF-IDF terms. One would then use those to calculate the cosine similarity of candidate PubMed records (outside LC) and use that to retrieve a suitable set of records that are highly related. Those could be presented *via* Dashboard.

## Data availability statement

The original contributions presented in the study are included in the article/Supplementary material, further inquiries can be directed to the corresponding author.

## Author contributions

AP coordinated the analyses and led the article drafting. MM developed and executed many of the core analyses. NN contributed insights and analytical guidance throughout the study. All authors contributed to the article and approved the submitted version.

## Footnotes

^1^Our country publication counts above are for our PubMed data; our country counts using WoS data would be the US (1,382); the UK (549); and Italy (572).

^2^*Cureus*, also known as the *Cureus Journal of Medical Science*, is an open-access general medical journal founded in 2009. Google finds a note that it doesn't promote the use of journal impact factors. As noted in Section 4.5.1, it is the top publication for LC research.

^3^Using the NLP routine in *VantagePoint* that is tailored to ST&I texts.

^4^We explored various *VantagePoint* clustering routines.

^5^In contrast, running PCA offers leeway in versions of PCA and choices concerning a number of factors to extract.

^6^As of December 2022, WoS indexes over 21,000 journals, 205,000 conference proceedings, and 104,000 books. The number of WoSCs increases gradually, with some 250 currently.

^7^Conferences are underrepresented because of the variability in naming and the inclusion of yearly variations in conference naming.

^8^Various JIFs can be computed; these also change over time periods. Those noted here were drawn from Google search. They should just be taken as suggestive of the degree of scientific influence.

^9^The data have not been fully consolidated. In particular, note acronyms and full phrases both occur, c.f., “PFT”.

^10^If we have missed any, blame it on ALP's color blindness.

^11^In Section 3.2, Data Treatment, there is a degree of noise in assigning monthly publication dates.

^12^Trying not to weight the first period heavily due to there being only 46 publications. Also note that 3,643 of the 5,243 publications are associated with one or more of the 13 topic clusters for these periods.

^13^Funding data coverage, even for the WoS set, is only 43%, so we don't have much to say. Applying only limited cleaning/consolidation, the US National Institutes of Health (NIH with 241 records), the National Natural Science Foundation of China (NSFC with 107), and the National Institute for Health Research (the UK with 97) are active supporters.
