# Supplementary material for: The long COVID research literature
Source: Front Res Metr Anal. 2023 Mar 24;8:1149091. doi: 10.3389/frma.2023.1149091 (PMC10080666; doi:10.3389/frma.2023.1149091)
Supplement: Supplementary file 1 [file Data_Sheet_1.docx]

**Supplemental Materials**

**Table S1. Web of Science Categories with Long COVID Publications**

| **LC Rank** | **Web of Science Categories** | **# Records** |
| --- | --- | --- |
| 1 | Medicine, General & Internal | 793 |
| 2 | Immunology | 338 |
| 3 | Public, Environmental & Occupational Health | 297 |
| 4 | Neurosciences | 287 |
| 5 | Clinical Neurology | 277 |
| 6 | Infectious Diseases | 255 |
| 7 | Medicine, Research & Experimental | 243 |
| 8 | Respiratory System | 218 |
| 9 | Psychiatry | 217 |
| 10 | Cardiac & Cardiovascular Systems | 192 |
| 11 | Pharmacology & Pharmacy | 166 |
| 12 | Microbiology | 161 |
| 13 | Biochemistry & Molecular Biology | 150 |
| 14 | Multidisciplinary Sciences | 147 |
| 15 | Pediatrics | 121 |
| 16 | Health Care Sciences & Services | 120 |
| 17 | Environmental Sciences | 116 |
| 18 | Virology | 105 |
| 19 | Cell Biology | 101 |
| 20 | Rehabilitation | 90 |
| 21 | Endocrinology & Metabolism | 87 |
| 22 | Radiology, Nuclear Medicine & Medical Imaging | 73 |
| 23 | Surgery | 60 |
| 24 | Sport Sciences | 57 |
| 25 | Nutrition & Dietetics | 56 |
| 26 | Critical Care Medicine | 52 |
| 27 | Health Policy & Services | 51 |
| 28 | Oncology | 51 |
| 29 | Geriatrics & Gerontology | 49 |
| 30 | Hematology | 49 |
| 31 | Physiology | 46 |
| 32 | Psychology, Clinical | 46 |
| 33 | Nursing | 42 |
| 34 | Peripheral Vascular Disease | 42 |
| 35 | Biology | 40 |
| 36 | Chemistry, Multidisciplinary | 37 |
| 37 | Otorhinolaryngology | 33 |
| 38 | Primary Health Care | 33 |
| 39 | Ophthalmology | 32 |
| 40 | Psychology | 32 |
| 41 | Urology & Nephrology | 32 |
| 42 | Gastroenterology & Hepatology | 28 |
| 43 | Rheumatology | 27 |
| 44 | Pathology | 26 |
| 45 | Dermatology | 25 |
| 46 | Orthopedics | 23 |
| 47 | Chemistry, Medicinal | 22 |
| 48 | Anesthesiology | 20 |
| 49 | Medical Informatics | 20 |
| 50 | Medical Laboratory Technology | 20 |
| 51 | Biotechnology & Applied Microbiology | 19 |
| 52 | Obstetrics & Gynecology | 19 |
| 53 | Psychology, Multidisciplinary | 19 |
| 54 | Tropical Medicine | 17 |
| 55 | Biophysics | 16 |
| 56 | Integrative & Complementary Medicine | 16 |
| 57 | Dentistry, Oral Surgery & Medicine | 15 |
| 58 | Parasitology | 15 |
| 59 | Biochemical Research Methods | 14 |
| 60 | Genetics & Heredity | 14 |
| 61 | Food Science & Technology | 13 |
| 62 | Transplantation | 13 |
| 63 | Chemistry, Analytical | 12 |
| 64 | Allergy | 11 |
| 65 | Gerontology | 10 |
| 66 | Emergency Medicine | 9 |
| 67 | Nanoscience & Nanotechnology | 9 |
| 68 | Engineering, Biomedical | 8 |
| 69 | Social Sciences, Biomedical | 8 |
| 70 | Toxicology | 8 |
| 71 | Behavioral Sciences | 7 |
| 72 | Substance Abuse | 7 |
| 73 | Cell & Tissue Engineering | 6 |
| 74 | Instruments & Instrumentation | 6 |
| 75 | Computer Science, Interdisciplinary Applications | 5 |
| 76 | Developmental Biology | 5 |
| 77 | Mathematical & Computational Biology | 5 |
| 78 | Neuroimaging | 5 |
| 79 | Psychology, Developmental | 5 |
| 80 | Reproductive Biology | 5 |
| 81 | Economics | 4 |
| 82 | Electrochemistry | 4 |
| 83 | Women's Studies | 4 |
| 84 | Andrology | 3 |
| 85 | Computer Science, Information Systems | 3 |
| 86 | Computer Science, Theory & Methods | 3 |
| 87 | Education, Scientific Disciplines | 3 |
| 88 | Engineering, Electrical & Electronic | 3 |
| 89 | Materials Science, Multidisciplinary | 3 |
| 90 | Mycology | 3 |
| 91 | Social Sciences, Interdisciplinary | 3 |
| 92 | Veterinary Sciences | 3 |
| 93 | Acoustics | 2 |
| 94 | Audiology & Speech-Language Pathology | 2 |
| 95 | Computer Science, Hardware & Architecture | 2 |
| 96 | Education & Educational Research | 2 |
| 97 | Ethics | 2 |
| 98 | History & Philosophy Of Science | 2 |
| 99 | Humanities, Multidisciplinary | 2 |
| 100 | Information Science & Library Science | 2 |
| 101 | Management | 2 |
| 102 | Medical Ethics | 2 |
| 103 | Medicine, Legal | 2 |
| 104 | Meteorology & Atmospheric Sciences | 2 |
| 105 | Agriculture, Dairy & Animal Science | 1 |
| 106 | Agriculture, Multidisciplinary | 1 |
| 107 | Anatomy & Morphology | 1 |
| 108 | Chemistry, Physical | 1 |
| 109 | Computer Science, Artificial Intelligence | 1 |
| 110 | Computer Science, Software Engineering | 1 |
| 111 | Education, Special | 1 |
| 112 | Engineering, Chemical | 1 |
| 113 | Family Studies | 1 |
| 114 | Language & Linguistics | 1 |
| 115 | Linguistics | 1 |
| 116 | Materials Science, Biomaterials | 1 |
| 117 | Optics | 1 |
| 118 | Physics, Applied | 1 |
| 119 | Plant Sciences | 1 |
| 120 | Psychology, Educational | 1 |
| 121 | Psychology, Experimental | 1 |
| 122 | Psychology, Psychoanalysis | 1 |
| 123 | Psychology, Social | 1 |
| 124 | Social Issues | 1 |
| 125 | Transportation | 1 |
| 126 | Zoology | 1 |


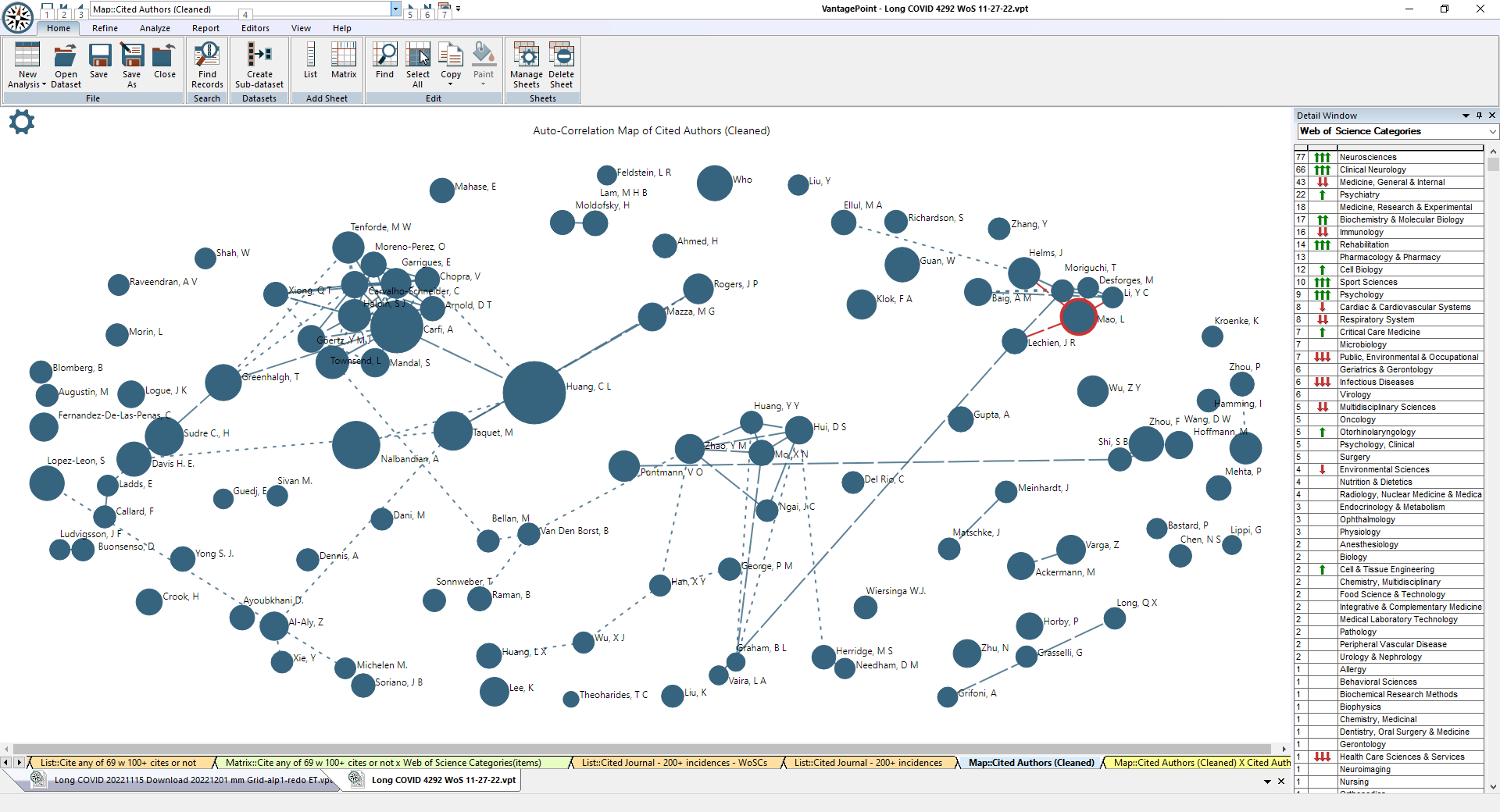


**Figure S1. Auto-correlation Map of 107 Highly Cited Authors**


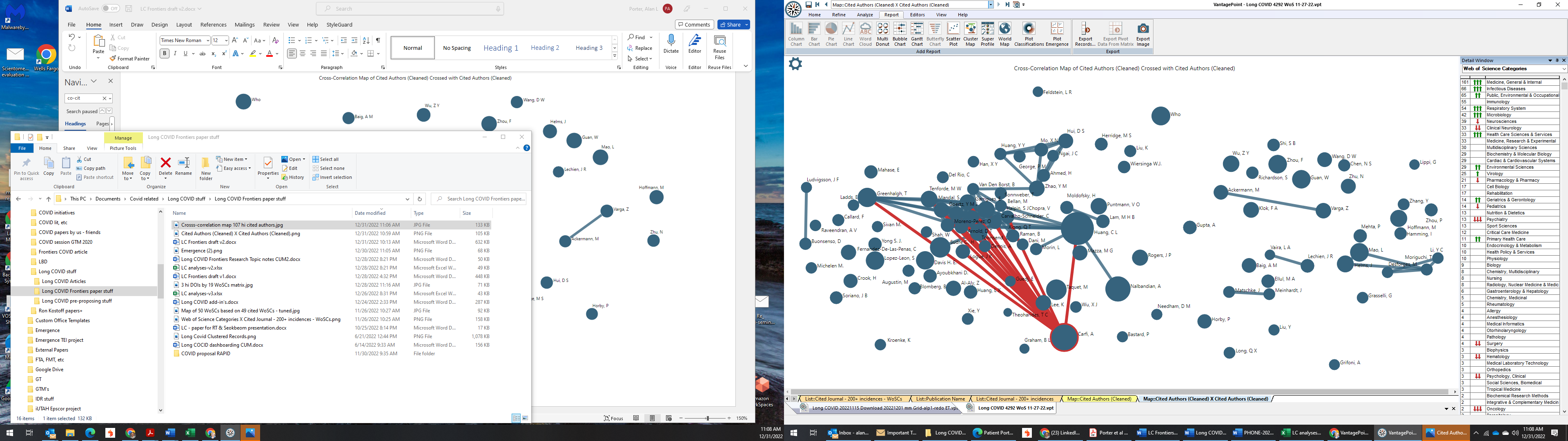


**Figure S2. Cross-correlation Map of 107 Highly Cited Author**

**Table S2. Countries vs. Long COVID Topic Clusters**

|  |  | 1 | 2 | 3 | 4 | 5 | 6 | 7 | 8 | 9 | 10 | 11 | 12 | 13 |
| --- | --- | --- | --- | --- | --- | --- | --- | --- | --- | --- | --- | --- | --- | --- |
|  | # Records | 1137 | 1092 | 1019 | 1016 | 896 | 769 | 747 | 632 | 628 | 575 | 371 | 135 | 130 |
| **# Records** | **Country \ Topic** | **Neuropsychiatric Symptoms** | **Mortality** | **Sequelae** | **Hospitalization** | **Fatigue** | **Disease Severity** | **Pathophysiology** | **Cognitive Deficits** | **Neurological Sequelae** | **Acute Phase** | **Lungs** | **Long-Term Effects** | **Fever** |
| 1317 | USA | 254 | 311 | 352 | 214 | 203 | 216 | 228 | 130 | 178 | 140 | 86 | 32 | 27 |
| 626 | Italy | 154 | 122 | 98 | 142 | 112 | 80 | 106 | 98 | 94 | 80 | 54 | 15 | 12 |
| 349 | China | 82 | 69 | 69 | 70 | 50 | 65 | 55 | 36 | 22 | 22 | 47 | 14 | 5 |
| 329 | Germany | 70 | 63 | 43 | 50 | 78 | 62 | 54 | 53 | 49 | 43 | 20 | 5 | 4 |
| 289 | Spain | 80 | 68 | 56 | 101 | 61 | 32 | 40 | 36 | 27 | 43 | 20 | 5 | 6 |
| 229 | India | 35 | 50 | 40 | 27 | 26 | 37 | 37 | 19 | 34 | 17 | 22 | 4 | 9 |
| 197 | Canada | 54 | 37 | 29 | 32 | 34 | 31 | 30 | 33 | 29 | 24 | 6 | 6 | 5 |
| 179 | France | 40 | 41 | 26 | 45 | 34 | 19 | 34 | 29 | 34 | 30 | 14 | 0 | 2 |
| 151 | UK | 32 | 43 | 25 | 25 | 33 | 27 | 25 | 29 | 25 | 23 | 10 | 3 | 2 |
| 144 | Brazil | 34 | 29 | 22 | 35 | 24 | 13 | 28 | 34 | 27 | 27 | 13 | 1 | 1 |
| 138 | Switzerland | 23 | 33 | 25 | 18 | 31 | 20 | 16 | 19 | 20 | 16 | 8 | 1 | 1 |
| 135 | Australia | 45 | 19 | 17 | 20 | 24 | 22 | 14 | 21 | 17 | 14 | 6 | 6 | 4 |
| 111 | Netherlands | 28 | 16 | 27 | 27 | 22 | 18 | 12 | 21 | 6 | 11 | 4 | 4 | 1 |
| 107 | Denmark | 26 | 24 | 14 | 41 | 29 | 14 | 21 | 18 | 16 | 15 | 10 | 5 | 4 |
| 99 | Japan | 19 | 17 | 23 | 22 | 16 | 14 | 14 | 17 | 18 | 11 | 5 | 0 | 8 |
| 83 | Turkey | 17 | 15 | 13 | 25 | 14 | 9 | 11 | 5 | 9 | 8 | 4 | 3 | 3 |
| 82 | Russia | 19 | 15 | 12 | 13 | 13 | 12 | 17 | 15 | 9 | 12 | 7 | 2 | 0 |
| 82 | Sweden | 17 | 13 | 14 | 15 | 18 | 12 | 10 | 10 | 14 | 12 | 1 | 3 | 3 |
| 77 | Belgium | 13 | 11 | 9 | 22 | 13 | 9 | 11 | 13 | 8 | 7 | 5 | 2 | 0 |
| 75 | Ireland | 22 | 11 | 10 | 16 | 15 | 5 | 7 | 12 | 1 | 9 | 5 | 0 | 0 |
| 72 | Greece | 15 | 15 | 14 | 13 | 17 | 19 | 11 | 8 | 5 | 10 | 2 | 2 | 3 |
| 72 | Iran | 15 | 19 | 9 | 11 | 11 | 6 | 15 | 4 | 11 | 15 | 3 | 0 | 2 |
| 71 | Austria | 15 | 15 | 13 | 12 | 20 | 11 | 7 | 12 | 9 | 2 | 4 | 0 | 0 |
| 68 | Poland | 14 | 23 | 5 | 10 | 14 | 13 | 12 | 7 | 11 | 18 | 2 | 1 | 0 |
| 67 | The Netherlands | 14 | 9 | 14 | 16 | 12 | 10 | 5 | 12 | 2 | 5 | 2 | 3 | 0 |
| 61 | Egypt | 12 | 21 | 6 | 6 | 11 | 13 | 8 | 8 | 11 | 5 | 3 | 1 | 1 |
| 58 | Israel | 10 | 12 | 8 | 11 | 16 | 9 | 12 | 9 | 5 | 5 | 2 | 0 | 2 |
| 54 | South Korea | 18 | 8 | 8 | 3 | 8 | 9 | 5 | 8 | 10 | 3 | 3 | 5 | 2 |
| 53 | Mexico | 9 | 12 | 13 | 12 | 12 | 7 | 12 | 6 | 12 | 3 | 6 | 2 | 3 |
| 50 | Saudi Arabia | 10 | 14 | 9 | 9 | 10 | 7 | 11 | 5 | 11 | 3 | 7 | 1 | 3 |
| 47 | Singapore | 11 | 10 | 4 | 3 | 6 | 6 | 8 | 4 | 5 | 5 | 2 | 0 | 1 |
| 47 | South Africa | 8 | 12 | 10 | 6 | 9 | 6 | 9 | 5 | 4 | 5 | 2 | 2 | 0 |
| 45 | Pakistan | 10 | 13 | 7 | 7 | 6 | 4 | 5 | 6 | 9 | 7 | 3 | 2 | 1 |
| 37 | Portugal | 10 | 12 | 7 | 10 | 7 | 5 | 5 | 7 | 7 | 1 | 3 | 0 | 1 |
| 36 | Deutschland | 9 | 5 | 8 | 3 | 8 | 10 | 4 | 8 | 2 | 5 | 3 | 1 | 0 |
| 34 | Norway | 7 | 6 | 6 | 11 | 11 | 6 | 4 | 6 | 4 | 3 | 2 | 0 | 0 |
| 32 | Chile | 8 | 6 | 5 | 12 | 3 | 2 | 4 | 5 | 4 | 3 | 4 | 0 | 1 |
| 31 | Malaysia | 9 | 4 | 1 | 2 | 10 | 3 | 4 | 5 | 3 | 3 | 3 | 1 | 0 |
| 29 | Romania | 4 | 11 | 4 | 8 | 3 | 2 | 7 | 1 | 0 | 5 | 5 | 2 | 1 |
| 24 | Indonesia | 4 | 2 | 2 | 6 | 5 | 5 | 6 | 1 | 2 | 1 | 2 | 0 | 1 |
| 24 | New Zealand | 7 | 9 | 5 | 6 | 1 | 6 | 3 | 3 | 1 | 2 | 1 | 0 | 1 |
| 23 | Taiwan | 6 | 3 | 5 | 3 | 3 | 4 | 5 | 1 | 5 | 0 | 3 | 2 | 0 |
| 22 | Bangladesh | 4 | 2 | 2 | 2 | 4 | 2 | 0 | 5 | 3 | 1 | 1 | 1 | 1 |
| 21 | Colombia | 7 | 1 | 3 | 2 | 5 | 0 | 2 | 6 | 5 | 3 | 0 | 0 | 0 |
| 20 | Croatia | 2 | 6 | 6 | 3 | 2 | 3 | 5 | 3 | 2 | 2 | 1 | 1 | 0 |
| 20 | Thailand | 5 | 6 | 2 | 1 | 4 | 4 | 4 | 0 | 3 | 4 | 2 | 1 | 0 |
| 19 | Czech Republic | 9 | 7 | 6 | 2 | 8 | 6 | 4 | 4 | 2 | 4 | 2 | 1 | 0 |
| 18 | Argentina | 6 | 1 | 4 | 3 | 4 | 3 | 2 | 2 | 5 | 6 | 0 | 0 | 1 |
| 17 | Nigeria | 4 | 4 | 4 | 5 | 3 | 1 | 5 | 2 | 2 | 2 | 2 | 0 | 0 |
| 16 | Qatar | 4 | 5 | 4 | 0 | 3 | 1 | 3 | 1 | 4 | 1 | 3 | 1 | 1 |
| 16 | North Korea | 5 | 2 | 1 | 1 | 1 | 3 | 1 | 2 | 2 | 1 | 1 | 0 | 0 |
| 16 | UAE | 2 | 7 | 3 | 4 | 4 | 1 | 3 | 4 | 0 | 3 | 3 | 0 | 0 |
| 15 | Georgia | 0 | 2 | 2 | 3 | 5 | 1 | 1 | 1 | 4 | 0 | 0 | 0 | 3 |
| 14 | Ecuador | 1 | 5 | 1 | 1 | 3 | 2 | 2 | 2 | 2 | 1 | 1 | 0 | 1 |
| 14 | Hungary | 2 | 3 | 3 | 3 | 2 | 4 | 2 | 0 | 2 | 3 | 0 | 0 | 0 |
| 14 | Iraq | 4 | 7 | 1 | 3 | 5 | 2 | 2 | 2 | 3 | 4 | 1 | 1 | 0 |
| 13 | Finland | 1 | 2 | 2 | 4 | 3 | 1 | 1 | 4 | 1 | 2 | 1 | 1 | 0 |
| 12 | Hong Kong | 6 | 4 | 2 | 3 | 1 | 0 | 1 | 2 | 0 | 0 | 1 | 2 | 0 |
| 12 | Peru | 2 | 2 | 2 | 2 | 1 | 1 | 0 | 1 | 1 | 1 | 2 | 0 | 2 |
| 11 | Bulgaria | 3 | 3 | 2 | 3 | 4 | 1 | 4 | 3 | 1 | 4 | 0 | 0 | 0 |
| 11 | Lebanon | 6 | 5 | 2 | 1 | 2 | 0 | 2 | 1 | 0 | 2 | 0 | 1 | 1 |
| 10 | Brasil | 2 | 2 | 3 | 3 | 2 | 2 | 0 | 1 | 1 | 0 | 2 | 0 | 0 |
| 10 | Slovakia | 1 | 2 | 1 | 0 | 2 | 4 | 4 | 1 | 1 | 2 | 0 | 0 | 0 |
| 9 | Jordan | 3 | 3 | 3 | 1 | 1 | 1 | 0 | 1 | 1 | 0 | 1 | 0 | 1 |
| 9 | Morocco | 3 | 4 | 1 | 1 | 0 | 1 | 0 | 0 | 0 | 2 | 1 | 0 | 0 |
| 9 | Nepal | 0 | 4 | 2 | 1 | 3 | 0 | 0 | 2 | 1 | 0 | 0 | 1 | 1 |
| 9 | Serbia | 2 | 4 | 2 | 1 | 0 | 2 | 3 | 1 | 1 | 0 | 0 | 0 | 0 |
| 9 | Vietnam | 5 | 4 | 1 | 3 | 1 | 1 | 2 | 1 | 2 | 1 | 1 | 0 | 0 |
| 8 | Oman | 1 | 4 | 1 | 1 | 2 | 1 | 1 | 0 | 0 | 2 | 1 | 0 | 0 |
| 8 | Philippines | 1 | 3 | 1 | 1 | 1 | 3 | 1 | 0 | 1 | 1 | 0 | 0 | 0 |
| 7 | Cyprus | 2 | 1 | 1 | 0 | 0 | 1 | 1 | 1 | 0 | 1 | 0 | 1 | 0 |
| 7 | Ghana | 7 | 0 | 1 | 1 | 0 | 1 | 0 | 1 | 0 | 1 | 0 | 0 | 0 |
| 6 | Faroe Islands | 0 | 0 | 0 | 0 | 3 | 1 | 0 | 1 | 2 | 5 | 1 | 0 | 0 |
| 6 | Kenya | 2 | 0 | 3 | 1 | 1 | 1 | 1 | 0 | 0 | 2 | 0 | 0 | 0 |
| 6 | Slovenia | 1 | 2 | 0 | 1 | 0 | 1 | 0 | 0 | 1 | 1 | 0 | 0 | 0 |
| 6 | Tunisia | 1 | 2 | 1 | 1 | 3 | 0 | 0 | 0 | 2 | 0 | 0 | 0 | 0 |
| 5 | Jersey | 0 | 1 | 0 | 2 | 0 | 1 | 0 | 0 | 0 | 2 | 0 | 0 | 0 |
| 5 | Luxembourg | 1 | 2 | 2 | 1 | 0 | 0 | 2 | 1 | 3 | 1 | 0 | 0 | 0 |
| 5 | U.K | 0 | 0 | 1 | 0 | 2 | 0 | 1 | 0 | 1 | 0 | 1 | 0 | 0 |
| 4 | Bosnia-Herzegovina | 2 | 2 | 1 | 1 | 0 | 0 | 1 | 1 | 3 | 1 | 0 | 0 | 0 |
| 4 | Costa Rica | 0 | 0 | 2 | 0 | 1 | 0 | 1 | 1 | 0 | 0 | 0 | 0 | 1 |
| 4 | Cuba | 3 | 0 | 1 | 1 | 2 | 1 | 2 | 3 | 2 | 1 | 0 | 1 | 0 |
| 4 | Ethiopia | 2 | 2 | 0 | 1 | 1 | 1 | 1 | 0 | 0 | 0 | 0 | 0 | 1 |
| 4 | Latvia | 0 | 0 | 1 | 1 | 1 | 0 | 0 | 0 | 2 | 1 | 0 | 0 | 1 |
| 4 | Malta | 1 | 2 | 0 | 1 | 1 | 1 | 0 | 0 | 1 | 0 | 0 | 0 | 0 |
| 4 | Ukraine | 1 | 3 | 0 | 1 | 0 | 2 | 1 | 2 | 1 | 0 | 0 | 0 | 0 |
| 3 | Bahrain | 1 | 0 | 0 | 0 | 1 | 0 | 0 | 0 | 2 | 1 | 0 | 0 | 0 |
| 3 | Estonia | 1 | 0 | 0 | 0 | 0 | 1 | 0 | 0 | 1 | 1 | 0 | 0 | 0 |
| 3 | Saudi Arabia | 2 | 1 | 2 | 0 | 0 | 0 | 1 | 1 | 1 | 0 | 0 | 0 | 0 |
| 3 | Mauritius | 1 | 1 | 0 | 0 | 1 | 2 | 1 | 0 | 1 | 0 | 0 | 0 | 0 |
| 3 | Sri Lanka | 1 | 1 | 0 | 0 | 1 | 1 | 2 | 0 | 1 | 0 | 0 | 0 | 1 |
| 3 | Sudan | 2 | 1 | 0 | 0 | 1 | 0 | 0 | 0 | 0 | 0 | 0 | 1 | 0 |
| 3 | Uganda | 2 | 1 | 1 | 0 | 0 | 1 | 2 | 1 | 2 | 1 | 0 | 0 | 0 |
| 3 | Zambia | 1 | 0 | 0 | 0 | 1 | 0 | 0 | 0 | 0 | 0 | 0 | 0 | 0 |
| 2 | Armenia | 0 | 0 | 0 | 1 | 1 | 1 | 0 | 0 | 2 | 0 | 0 | 0 | 0 |
| 2 | Bolivia | 1 | 0 | 0 | 0 | 0 | 0 | 1 | 2 | 1 | 1 | 0 | 0 | 0 |
| 2 | Grenada | 0 | 0 | 0 | 1 | 0 | 0 | 1 | 0 | 0 | 0 | 1 | 0 | 0 |
| 2 | Jamaica | 0 | 1 | 1 | 1 | 0 | 0 | 1 | 0 | 0 | 0 | 0 | 0 | 0 |
| 2 | Kazakhstan | 0 | 1 | 0 | 1 | 0 | 1 | 1 | 0 | 0 | 0 | 0 | 0 | 0 |
| 2 | Kosovo | 0 | 2 | 0 | 0 | 0 | 0 | 0 | 0 | 0 | 1 | 0 | 0 | 0 |
| 2 | Macedonia | 0 | 1 | 1 | 0 | 0 | 0 | 2 | 0 | 1 | 0 | 0 | 0 | 0 |
| 2 | Panama | 0 | 1 | 1 | 2 | 0 | 1 | 1 | 0 | 0 | 0 | 0 | 0 | 0 |
| 2 | Paraguay | 0 | 0 | 1 | 0 | 1 | 0 | 0 | 0 | 0 | 0 | 0 | 0 | 1 |
| 2 | Saint Kitts and Nevis | 1 | 1 | 0 | 0 | 1 | 0 | 1 | 0 | 0 | 1 | 1 | 0 | 0 |
| 2 | Yemen | 0 | 0 | 0 | 0 | 0 | 0 | 0 | 0 | 1 | 0 | 0 | 0 | 0 |
| 1 | Afghanistan | 0 | 1 | 0 | 0 | 0 | 0 | 0 | 0 | 1 | 0 | 0 | 1 | 0 |
| 1 | Anguilla | 1 | 1 | 0 | 0 | 1 | 0 | 1 | 0 | 0 | 1 | 1 | 0 | 0 |
| 1 | Azerbaijan | 0 | 0 | 1 | 0 | 0 | 0 | 0 | 0 | 0 | 1 | 0 | 0 | 0 |
| 1 | Bahamas | 0 | 0 | 0 | 0 | 0 | 0 | 0 | 0 | 0 | 0 | 0 | 0 | 0 |
| 1 | Barbados | 0 | 0 | 0 | 0 | 0 | 0 | 0 | 0 | 0 | 0 | 0 | 0 | 0 |
| 1 | Belarus | 0 | 0 | 0 | 0 | 0 | 0 | 0 | 0 | 0 | 0 | 0 | 0 | 0 |
| 1 | Burkina Faso | 1 | 0 | 1 | 0 | 0 | 0 | 0 | 1 | 0 | 0 | 0 | 0 | 0 |
| 1 | Cambodia | 1 | 0 | 1 | 0 | 0 | 0 | 0 | 1 | 0 | 0 | 0 | 0 | 0 |
| 1 | Cameroon | 0 | 0 | 0 | 0 | 0 | 0 | 0 | 0 | 0 | 0 | 0 | 0 | 0 |
| 1 | Congo | 0 | 0 | 0 | 0 | 0 | 0 | 0 | 0 | 0 | 0 | 0 | 0 | 0 |
| 1 | Cote d'Ivoire | 1 | 0 | 0 | 0 | 0 | 0 | 0 | 0 | 0 | 0 | 0 | 0 | 0 |
| 1 | Dominica | 1 | 1 | 0 | 0 | 1 | 0 | 1 | 0 | 0 | 1 | 1 | 0 | 0 |
| 1 | Gabon | 0 | 1 | 1 | 1 | 0 | 0 | 0 | 0 | 0 | 0 | 0 | 0 | 0 |
| 1 | Guadeloupe | 1 | 0 | 0 | 0 | 0 | 0 | 0 | 1 | 1 | 1 | 0 | 0 | 0 |
| 1 | Haiti | 1 | 0 | 0 | 0 | 0 | 0 | 1 | 1 | 1 | 1 | 0 | 0 | 0 |
| 1 | Iceland | 1 | 0 | 0 | 0 | 0 | 0 | 0 | 0 | 0 | 1 | 0 | 0 | 0 |
| 1 | Kuwait | 0 | 0 | 0 | 0 | 0 | 1 | 0 | 0 | 0 | 0 | 0 | 0 | 0 |
| 1 | Libya | 0 | 0 | 0 | 1 | 0 | 0 | 0 | 0 | 0 | 0 | 0 | 0 | 0 |
| 1 | Liechtenstein | 0 | 0 | 0 | 0 | 0 | 0 | 0 | 1 | 0 | 0 | 0 | 0 | 0 |
| 1 | Lithuania | 0 | 0 | 0 | 0 | 1 | 0 | 0 | 0 | 0 | 0 | 1 | 0 | 0 |
| 1 | Martinique | 0 | 0 | 0 | 1 | 0 | 0 | 0 | 0 | 0 | 0 | 0 | 0 | 0 |
| 1 | Monaco | 0 | 0 | 0 | 0 | 0 | 0 | 0 | 0 | 0 | 0 | 0 | 0 | 0 |
| 1 | Myanmar | 0 | 0 | 0 | 0 | 0 | 0 | 0 | 0 | 0 | 0 | 0 | 0 | 0 |
| 1 | Namibia | 0 | 0 | 0 | 0 | 0 | 0 | 0 | 0 | 0 | 0 | 0 | 0 | 0 |
| 1 | North Macedonia | 0 | 0 | 0 | 0 | 0 | 0 | 1 | 0 | 1 | 0 | 0 | 0 | 0 |
| 1 | Monaco | 0 | 0 | 0 | 0 | 0 | 0 | 0 | 0 | 0 | 0 | 0 | 0 | 0 |
| 1 | Singapore | 0 | 0 | 0 | 0 | 0 | 1 | 0 | 0 | 0 | 0 | 0 | 0 | 0 |
| 1 | Slovenia | 1 | 0 | 0 | 1 | 0 | 0 | 0 | 0 | 0 | 0 | 0 | 0 | 0 |
| 1 | Senegal | 0 | 1 | 1 | 0 | 0 | 1 | 1 | 0 | 1 | 0 | 0 | 0 | 0 |
| 1 | Sierra Leone | 1 | 0 | 1 | 1 | 0 | 0 | 0 | 1 | 0 | 1 | 0 | 0 | 0 |
| 1 | Somalia | 0 | 0 | 0 | 0 | 0 | 0 | 0 | 0 | 0 | 0 | 0 | 0 | 0 |
| 1 | Uruguay | 0 | 1 | 0 | 0 | 1 | 0 | 0 | 0 | 0 | 0 | 0 | 0 | 0 |
| 1 | Venezuela | 0 | 0 | 0 | 0 | 0 | 0 | 0 | 0 | 0 | 1 | 0 | 0 | 0 |
| 1 | Viet Nam | 0 | 0 | 0 | 0 | 1 | 0 | 1 | 0 | 0 | 0 | 0 | 0 | 0 |
| 1 | Zimbabwe | 0 | 0 | 0 | 0 | 0 | 0 | 0 | 0 | 0 | 0 | 0 | 0 | 0 |
